# Ologs: A Categorical Framework for Knowledge Representation

**DOI:** 10.1371/journal.pone.0024274

**Published:** 2012-01-31

**Authors:** David I. Spivak, Robert E. Kent

**Affiliations:** 1 Mathematics Department, Massachusetts Institute of Technology, Cambridge, Massachusetts, United States of America; 2 Ontologos, Pullman, Washington, United States of America; The Cochrane Collaboration, Germany

## Abstract

In this paper we introduce the olog, or ontology log, a category-theoretic model for knowledge representation (KR). Grounded in formal mathematics, ologs can be rigorously formulated and cross-compared in ways that other KR models (such as semantic networks) cannot. An olog is similar to a relational database schema; in fact an olog can serve as a data repository if desired. Unlike database schemas, which are generally difficult to create or modify, ologs are designed to be user-friendly enough that authoring or reconfiguring an olog is a matter of course rather than a difficult chore. It is hoped that learning to author ologs is much simpler than learning a database definition language, despite their similarity. We describe ologs carefully and illustrate with many examples. As an application we show that any primitive recursive function can be described by an olog. We also show that ologs can be aligned or connected together into a larger network using functors. The various methods of information flow and institutions can then be used to integrate local and global world-views. We finish by providing several different avenues for future research.

## 1 Introduction

Scientists have a pressing need to organize their experiments, their data, their results, and their conclusions into a framework such that this work is reusable, transferable, and comparable with the work of other scientists. In this paper, we will discuss the “ontology log” or *olog* as a possibility for such a framework. Ontology is the study of what something *is*, i.e the nature of a given subject, and ologs are designed to record the results of such a study. The structure of ologs is based on a branch of mathematics called category theory. An olog is roughly a category that models a given real-world situation.

The main advantages of authoring an olog rather than writing a prose description of a subject are that

- an olog gives a precise formulation of a conceptual world-view,
- an olog can be formulaically converted into a database schema,
- an olog can be extended as new information is obtained,
- an olog written by one author can be easily and precisely referenced by others,
- an olog can be input into a computer and “meaningfully stored”, and
- different ologs can be compared by functors, which in turn generate automatic terminology translation systems.

The main disadvantage to using ologs over prose, aside from taking more space on the page, is that writing a good olog demands a clarity of thought that ordinary writing or conversation can more easily elide. However, the contemplation required to write a good olog about a subject may have unexpected benefits as well.

A category is a mathematical structure that appears much like a directed graph: it consists of objects (often drawn as nodes or dots, but here drawn as boxes) and arrows between them. The feature of categories that distinguishes them from graphs is the ability to declare an equivalence relation on the set of paths. A functor is a mapping from one category to another that preserves the structure (i.e., the nodes, the arrows, and the equivalences). If one views a category as a kind of language (as we shall in this paper) then a functor would act as a kind of translating dictionary between languages. There are many good references on category theory, including [1], [2], [3], [4], [5], and [6]; the first and second are suited for general audiences, the third and fourth are suited for computer scientists, and the fifth and sixth are suited for mathematicians (in each class the first reference is easier than the second).

A basic olog, defined in Section 2, is a category in which the objects and arrows have been labeled by English-language phrases that indicate their intended meaning. The objects represent types of things, the arrows represent functional relationships (also known as aspects, attributes, or observables), and the commutative diagrams represent facts. **Figure 1** is a simple olog about an amino acid called arginine ([7]).
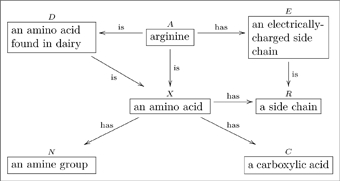
(1)


The idea of representing information in a graph is not new. For example the Resource Descriptive Framework (RDF) is a system for doing just that [8]. The key difference between a category and a graph is the consideration of paths, and that two paths from 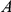 to 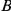 may be declared identical in a category. For example, we can further declare that in Diagram (1), the diagram
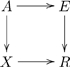
(2)
*commutes*, i.e., that the two paths 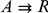 are equivalent, which can be translated as follows. Let 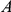 be a molecule of arginine. On the one hand 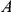, being an amino acid, has a side chain; on the other hand 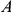 has an electrically-charged side-chain, which is of course a side chain. We seem to have associated *two* side-chains to 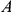, but in fact they both refer to the same physical thing, the same side-chain. Thus, the two paths 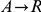 are deemed equivalent. The fact that this equivalence may seem trivial is not an indictment of the category idea but instead reinforces its importance – we must be able to indicate obvious facts within a given situation because what is obvious is the most essential.

While many situations can be modeled using basic ologs (categories), we often need to encode more structure. For this we will need so-called sketches. An olog will be defined as a finite limit, finite colimit sketch (see [9]), meaning we have the ability to encode objects (“types”), arrows (“aspects”), commutative diagrams (“facts”), as well as finite limits (“layouts”) and finite colimits (“groupings”).

Throughout this paper, whenever we refer to “the author” of an olog we are referring to the fictitious person who created it. We will refer to ourselves, David Spivak and Robert Kent, as “we” so as not to confuse things.

**1.0.1 Warning.** The author of an olog has a world-view, some fragment of which is captured in the olog. When person A examines the olog of person B, person A may or may not “agree with it.” For example, person B may have the following olog 
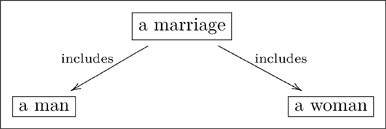
(3)which associates to each marriage a man and a woman. Person A may take the position that some marriages involve two men or two women, and thus see B’s olog as “wrong.” Such disputes are not “problems” with either A’s olog or B’s olog, they are discrepancies between world-views. Hence, throughout this paper, a reader R may see a displayed olog and notice a discrepancy between R’s world-view and our own, but R should not worry that this is a problem. This is not to say that ologs need not follow rules, but instead that the rules are enforced to ensure that an olog is structurally sound, rather than that it “correctly reflects reality,” whatever that may mean.

### 1.1 Plan of this paper

In this paper, we will define ologs and give several examples. We will state some rules of “good practice” which help one to author ologs that are meaningful to others and easily extendable. We will begin in Section 2 by laying out the basics: types as objects, aspects as arrows, and facts as commutative diagrams. In Section 3, we will explain how to attach “instance data” to an olog and hence realize ologs as database schemas. In Section 4, we will discuss meaningful constraints betweeen ologs that allow us to develop a higher-dimensional web of information called an information system, and we will discuss how the various parts of such a system interact via information channels. In Sections 5 and 6, we will extend the olog definition language to include “layouts” and “groupings”, which make for more expressive ologs; we will also describe two applications, one which explicates the computation of the factorial function, and the other which defines a notion from pure mathematics (that of pseudo-metric spaces). Finally, in Section 7, we will discuss some possible directions for future research.

For the remainder of the present section, we will explain how ologs relate to existing ideas in the field of knowledge representation.

### 1.2 The semantic advantage of ologs: modularity

The difference between ologs and prose is modularity: small conceptual pieces can form large ideas, and these pieces work best when they are reusable. The same phenomenon is true throughout computer science and mathematics. In programming languages, modularity brings not only vast efficiency to the writing of programs but enables an “abstraction barrier” that keeps the ideas clean. In mathematics, the most powerful results are often simple lemmas that are reusable in a wide variety of circumstances.

Web pages that consist of prose writing are often referred to as *information silos.* The idea is that a silo is a “big tube of stuff” which is not organized in any real way. Links between web pages provide some structure, but such a link does not carry with it a precise method to correlate the information within the two pages. Similarly in science, one author may reference another paper, but such a reference carries very little structure – it just points to a silo.

Ologs can be connected with links which are much richer than the link between two silos could possibly be. Individual concepts and connections within one olog can be “functorially aligned” with concepts and connections in another. A functor creates a precise connection between the work of one author and the work of another so that the precise nature of the comparison is not left to the reader’s imagination but explicitly specified. The ability to incorporate mathematical precision into the sharing of ideas is a central feature of ologs.

### 1.3 Relation to other models

There are many languages for knowledge representation (KR). For example, there are database languages such as SQL, ontology languages such as RDF and OWL, the language of Semantic Nets, and others (see [10]). One may ask what makes the olog concept different or better than the others.

The first response is that ologs are closely related to the above ideas. Indeed, all of these KR models can be “categorified” (i.e., phrased in the language of category theory) and related by functors, so that many of the ideas align and can be transferred between the different systems. In fact, as we will make clear in Section 3, ologs are almost identical to the categorical model of databases presented in Spivak’s unpublished paper “Functorial Data Migration” available online http://arxiv.org/abs/1009.1166, hereafter abbreviated FDM.

However, ologs have advantages over many existing KR models. The first advantage arises from the notion of commutative diagrams (which allow us to equate different paths through the domain, see Section 2.3) and of limits and colimits (which allow us to lay out and group things, see Sections 5 and 6). The additional expressivity of ologs give them a certain semantic clarity and interoperability that cannot be achieved with graphs and networks in the usual sense. The second advantage arises from the notion of olog morphisms, which allow the definition of meaningful constraints between ologs. With this in hand, we can integrate a set of similar ologs into a single information system, and go on to define information fusion. This will be discussed further Section 4.

In the remainder of this section we will provide a few more details on the relationship between ologs and each of the above KR models: databases, RDF/OWL, and semantic nets. The reader who does not know or care much about other systems of knowledge representation can skip to Section 2.

#### 1.3.1 Ologs and Databases

A database is a system of tables, each table of which consists of a header of columns and a set of rows. A table represents a type of thing 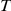, each column represents an attribute of 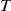, and each row represents an example of 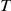. An attribute is itself a “type of thing”, so each column of a table points to another table.

The relationship between ologs and databases is that every box 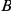 in an olog represents a type of thing and every arrow 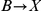 emanating from 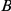 represents an attribute of 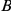 (whose results are of type 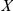). Thus the boxes and arrows in an olog correspond to tables and their columns in a database. The rows of each table in a database will correspond to “instances” of each type in an olog. Again, this will be made more clear in Section 3 or one can see FDM.

The point is that every olog can serve as a database schema, and the schemas represented by ologs range from simple (just objects and arrows) to complex (including commutative diagrams, products, sums, etc.). However, whereas database schemas are often prescriptive (“you must put your data into this format!”), ologs are usually descriptive (“this is how I see things”). One can think of an olog as an interface between people and databases: an olog is human readable, but it is also easily converted to a database schema upon which powerful applications can be put to work. Of course, if one is to use an olog as a database schema, it will become prescriptive. However, since the intention of each object and arrow is well-documented (as its label), schema evolution would be straightforward. Moreover, the categorical structure of ologs allows for *functorial data migration* by which one can transfer the instance data from an older schema to the current one (see FDM).

#### 1.3.2 Ologs and RDF/OWL

In FDM, the first author explained how a categorical database can be converted into an RDF triple store using the Grothendieck construction. The main difference between a categorical database schema (or an olog) and an RDF schema is that one cannot specify commutativity in an RDF schema. Thus one cannot express things like “the woman parent of a person 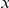 is the mother of 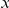.” Without this expressivity, it is hard to enforce much rigor, and thus RDF data tends to be too loose for many applications.

OWL schemas, on the other hand, can express many more constraints on classes and properties. We have not yet explored the connection, nor compared the expressive power, of ologs and OWL. However, they are significantly different systems, most obviously in that OWL relies on logic where ologs rely on category theory.

#### 1.3.3 Semantic Nets

On the surface, ologs look the most like semantic networks, or concept webs, but there are important differences between the two notions. First, arrows in a semantic network need not indicate functions; they can be relations. So there could be an arrow 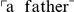 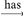 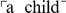 in a semantic network, but not in an olog (see Section 2.2.3 for how the same idea is expressible in an olog). There is a nice category of sets and relations, often denoted **Rel**, but this category is harder to reason about than is the ordinary category of sets and functions (often denoted 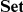). Thus, as mentioned above, semantic networks are categorifiable (using **Rel**), but this underlying formalism does not appear to play a part in the study or use of semantic networks. However, some attempt to integrate category theory and neural nets has been made, see [11].

Moreover, commutative diagrams and other expressive abilities held by ologs are not generally part of the semantic network concept (see [12]). For these reasons, semantic networks tend to be brittle: minor changes can have devastating effects. For example, if two semantic networks are somehow synced up and then one is changed, the linkage must be revised or may be altogether broken. Such a disaster is often avoided if one uses categories: because different paths can be equivalent, one can simply add new ideas (types and aspects) without changing the semantic meaning of what was already there. As Section 4.4 demonstates with an extended example, conceptual graphs, which are a popular formalism for semantics nets, can be linearized to ologs, thereby gaining in precision and expressibility.

## 2 Types, aspects, and facts

In this section we will explain basic ologs, which involve types, aspects, and facts. A basic olog is a category in which each object and arrow has been labeled by text; throughout this paper we will assume that text to be written in English.

The purpose of this section is to show how one can convert a real-world situation into an olog. It is probably impossible to explain this process precisely in words. Instead, we will explain mainly by example. We will give “rules of good practice” that lead to good ologs. While these rules are not strictly necessary, they help to ensure that the olog is properly formulated. As the Dalai Lama says, “Learn the rules so you know how to break them properly.”

### 2.1 Types

A type is an abstract concept, a distinction the author has made. We represent each type as a box containing a *singular indefinite noun phrase.* Each of the following four boxes is a type:
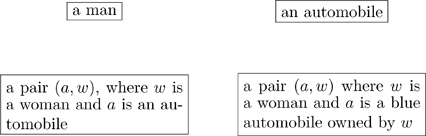
(4)


Each of the four boxes in (4) represents a type of thing, a whole class of things, and the label on that box is what one should call *each example* of that class. Thus 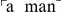 does not represent a single man, but the set of men, each example of which is called “a man”. Similarly, the bottom right-hand box in (4) represents an abstract type of thing, which probably has more than a million examples, but the label on the box indicates a common name for each such example.

Typographical problems emerge when writing a text-box in a line of text, e.g. the text-box <$>\scale 87%\raster="rg25"<$> seems out of place here, and the more in-line text-boxes one has in a given paragraph, the worse it gets. To remedy this, we will denote types which occur in a line of text with corner-symbols, e.g. we will write 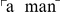 instead of 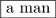.

For experts, types in ologs are intentional, rather than extensional – the label on a type describes its intention. The extension of a type will be captured by *instance data*; see Section 3

#### 2.1.1 Types with compound structures

Many types have compound structures; i.e., they are composed of smaller units. Examples include 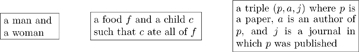
(5)


It is good practice to declare the variables in a “compound type”, as we did in the last two cases of (5). In other words, it is preferable to replace the first box above with something like 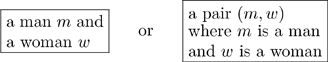
(6)so that the variables 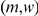 are clear.

#### 2.1.2 Rules of good practice

A type is presented as a text box. The text in that box should

i. begin with the word “a” or “an”;

ii. refer to a distinction made and recognizable by the author;

iii. refer to a distinction for which instances can be documented;

iv. not end in a punctuation mark;

v. declare all variables in a compound structure.

The first, second, and third rules ensure that the class of things represented by each box appears to the author as a well-defined set; see Section 3 for more details. The fourth and fifth rules encourage good “readability” of arrows, as will be discussed next in Section 2.2.

We will not always follow the rules of good practice throughout this document. We think of these rules being followed “in the background” but that we have “nicknamed” various boxes. So 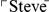 may stand as a nickname for 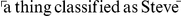 and 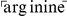 as a nickname for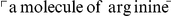.

### 2.2 Aspects

An aspect of a thing 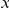 is a way of viewing it, a particular way in which 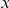 can be regarded or measured. For example, a woman can be regarded as a person; hence “being a person” is an aspect of a woman. A man has a height (say, taken in inches), so “having a height (in inches)” is an aspect of a man. In an olog, an aspect of 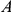 is represented by an arrow 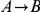, where 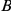 is the set of possible “answers” or results of the measurement. For example when observing the height of a man, the set of possible results is the set of integers, or perhaps the set of integers between 20 and 120.
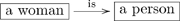
(7)

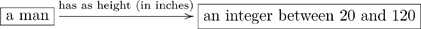
(8)


We will formalize the notion of aspect by saying that aspects are functional relationships. (Note that in type theory, what we here call aspects are called *functions*. Since our types are not fixed sets (see Section 3), we preferred a term that was less formal, namely “aspects”.) Suppose we wish to say that a thing classified as 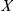 has an aspect 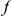 whose result set is 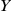. This means there is a functional relationship called 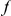 between 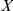 and 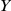, which can be denoted 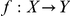. We call 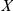 the *domain of definition* for the aspect 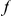, and we call 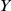 the *set of result values* for 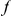. For example, a man has a height in inches whose result is an integer, and we could denote this by 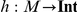. Here, 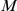 is the domain of definition for height and 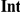 is the set of result values.

A set may always be drawn as a blob with dots in it. If 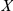 and 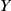 are two sets, then a *a function from* 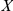 *to* 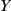, denoted 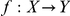 can be presented by drawing arrows from dots in blob 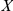 to dots in blob 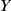. There are two rules:

i. each arrow must emanate *from* a dot in 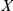 and point *to* a dot in 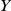;

ii. each dot in 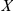 must have precisely *one* arrow emanating from it.

Given an element 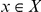, the arrow emanating from it points to some element 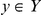, which we call *the image of* 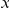 *under* 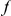 and denote 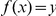.

Again, in an olog, an aspect of a thing 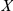 is drawn as a labeled arrow pointing from 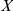 to a “set of result values.” Let us concentrate briefly on the arrow in (7). The domain of definition is the set of women (a set with perhaps 3 billion elements); the set of result values is the set of persons (a set with perhaps 6 billion elements). We can imagine drawing an arrow from each dot in the “woman” set to a unique dot in the “person” set. No woman points to two different people, nor to zero people – each woman is exactly one person – so the rules for a functional relationship are satisfied. Let us now concentrate briefly on the arrow in (8). The domain of definition is the set of men, the set of result values is the set of integers 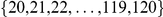. We can imagine drawing an arrow from each dot in the “man” set to a single dot in the “integer” set. No man points to two different heights, nor can a man have no height: each man has exactly one height. Note however that two different men can point to the same height.

#### 2.2.1 Invalid aspects

We tried above to clarify what it is that makes an aspect “valid”, namely that it must be a “functional relationship.” In this subsection we will present two arrows which on their face may appear to be aspects, but which on closer inspection are not functional (and hence are not valid as aspects).

Consider the following two arrows:
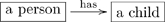
(7*)

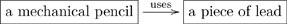
(8*)


A person may have no children or may have more than one child, so the first arrow is invalid: it is not functional because it does not satisfy rule (2) above. Similarly, if we drew an arrow from each mechanical pencil to each piece of lead it uses, it would not satisfy rule (2) above. Thus neither of these is a valid aspect.

Of course, in keeping with Warning 1.0.1, the above arrows may not be wrong but simply reflect that the author has a strange world-view or a strange vocabulary. Maybe the author believes that every mechanical pencil uses exactly one piece of lead. If this is so, then 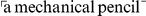 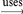 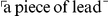 is indeed a valid aspect! Similarly, suppose the author meant to say that each person *was once* a child, or that a person has an inner child. Since every person has one and only one inner child (according to the author), the map 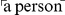 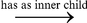 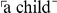 is a valid aspect. We cannot fault the author for such a view, but note that we have changed the name of the label to make its intention more explicit.

#### 2.2.2 Reading aspects and paths as English phrases

Each arrow (aspect) 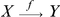 can be read by first reading the label on its source box (domain of definition) 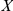, then the label on the arrow 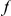, and finally the label on its target box (set of result values) 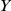. For example, the arrow
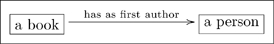
(9)is read “a book has as first author a person”, a valid English sentence.

Sometimes the label on an arrow can be shortened or dropped altogether if it is obvious from context. We will discuss this more in Section 2.3 but here is a common example from the way we write ologs.


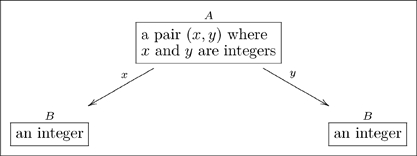
(10)

Neither arrow is readable by the protocol given above (e.g. “a pair 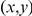 where 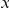 and 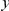 are integers 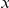 an integer” is not an English sentence), and yet it is obvious what each map means. For example, given the pair 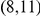 which belongs in box 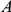, application of arrow 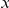 would yield 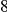 in box 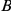. The label 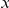 can be thought of as a nickname for the full name “yields, via the value of 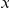,” and similarly for 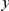. We do not generally use the full name for fear that the olog would become cluttered with text.

One can also read paths through an olog by inserting the word “which” after each intermediate box. For example the following olog has two paths of length 3 (counting arrows in a chain):
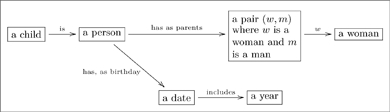
(11)


The top path is read “a child is a person, which has as parents a pair 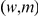 where 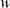 is a woman and 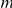 is a man, which yields, via the value of 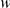, a woman.” The reader should read and understand the content of the bottom path.

#### 2.2.3 Converting non-functional relationships to aspects

There are many relationships that are not functional, and these cannot be considered aspects. Often the word “has” indicates a relationship – sometimes it is functional as in 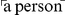 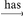 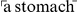, and sometimes it is not, as in 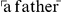 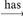 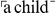. (Obviously, a father may have more than one child.) A quick fix would be to replace the latter by 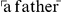 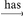 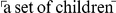. This is ok, but the relationship between 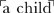
and 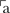 set of 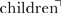
then becomes an issue to deal with later. There is another way to indicate such “non-functional” relationships.

In mathematics, a relation between sets 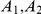, and so on through 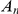 is defined to be a subset of the Cartesian product
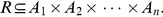
(12)


The set 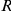 represents those sequences 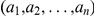 that are so-related. In an olog, we represent this as follows
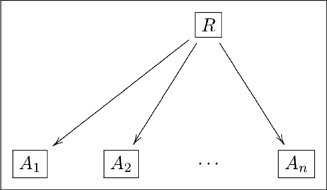
(13)


For example,



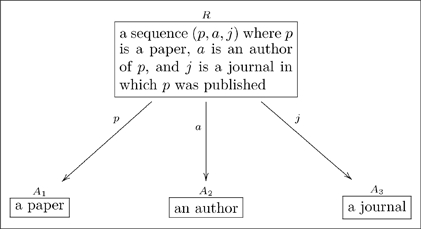
(14)Whereas 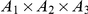 includes all possible triples 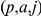 where 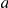 is a person, 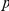 is a paper, and 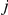 is a journal, it is obvious that not all such triples are found in 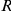. Thus 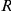 represents a proper subset of 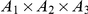.

*Rules of good practice* 2.1.2. An aspect is presented as a labeled arrow, pointing from a source box to a target box. The arrow text should

i. begin with a verb;

ii. yield an English sentence, when the source-box text followed by the arrow text followed by the target-box text is read;

iii. refer to a functional dependence: each instance of the source type should give rise to a specific instance of the target type;

### 2.3 Facts

In this section we will discuss facts and their relationship to “path equivalences.” It is such path equivalences, which exist in categories but do not exist in graphs, that make category theory so powerful.

Given an olog, the author may want to declare that two paths are equivalent. For example consider the two paths from 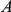 to 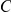 in the olog
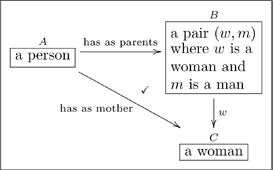
(15)


We know as English speakers that a woman parent is called a mother, so these two paths 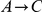 should be equivalent. A more mathematical way to say this is that the triangle in Olog (15) *commutes*.

A *commutative diagram* is a graph with some declared path equivalences. In the example above we concisely say “a woman parent is equivalent to a mother.” We declare this by defining the diagonal map in (15) to be *the composition* of the horizontal map and the vertical map.

We generally prefer to indicate a commutative diagram by drawing a check-mark, 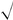, in the region bounded by the two paths, as in Olog (15). Sometimes, however, one cannot do this unambiguously on the 2-dimensional page. In such a case we will indicate the commutative diagrams (fact) by writing an equation. For example to say that the diagram
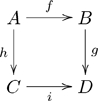
(16)


commutes, we could either draw a checkmark inside the square or write the equation 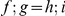 above it. Either way, it means that “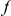 then 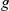
” is equivalent to “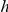 then 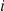
”.

**2.3.1 More complex facts.** Recording real-world facts in an olog can require some creativity. Whereas a fact like “the brother of ones father is ones uncle” is recorded as a simple commutative diagram, others are not so simple. We will try to show the range of expressivity of commutative diagrams in the following two examples.

#### 2.3.2 Example

How would one record a fact like “a truck weighs more than a car”? We suggest something like this:
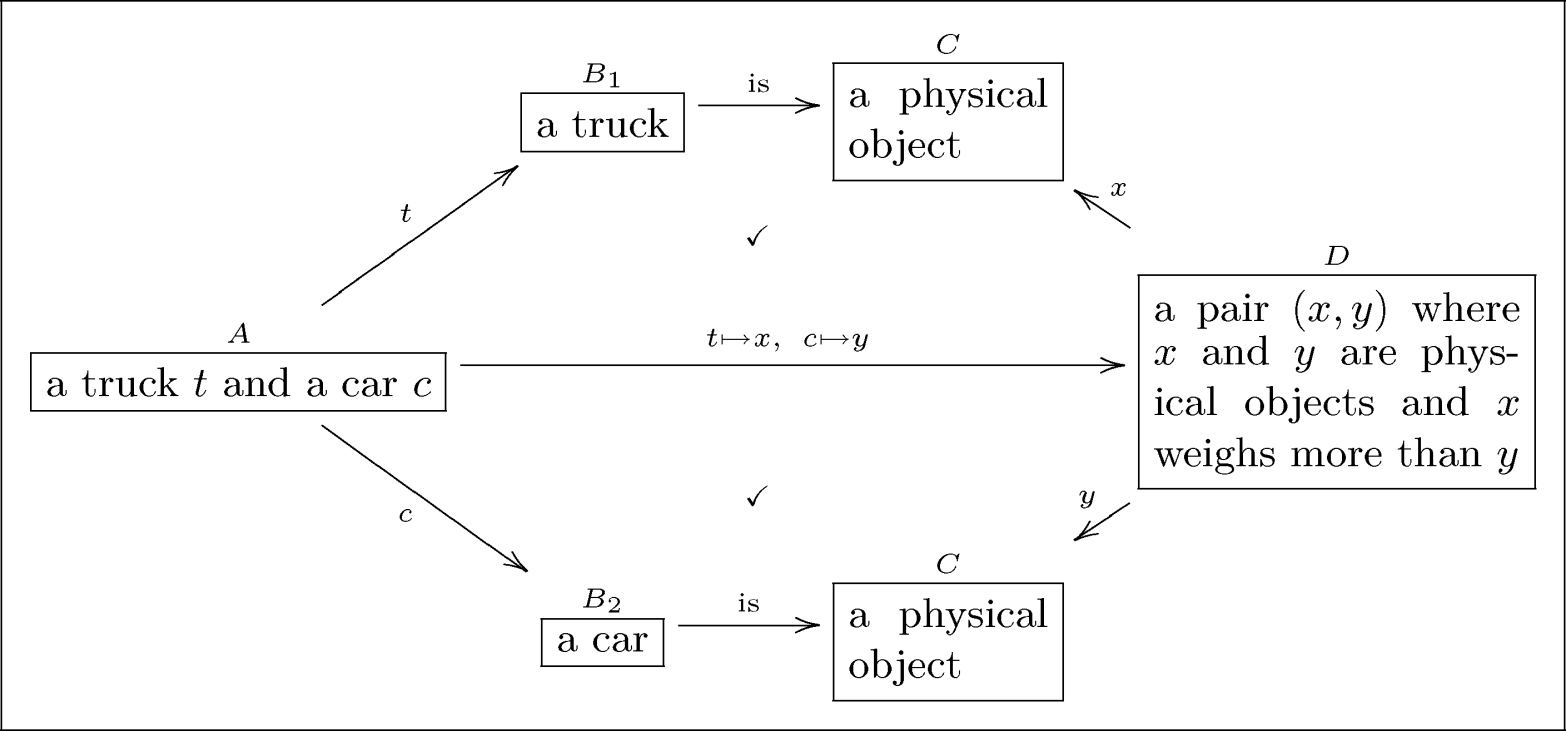
(17)


where both top and bottom commute. This olog exemplifies the fact that simple sentences sometimes contain large amounts of information. While the long map may seem to suffice to convey the idea “a truck weighs more than a car,” the path equivalences (declared by check-marks) serve to ground the idea in more basic types. These other types tend to be useful for other purposes, both within the olog and when connecting it to others.

#### 2.3.3 Specific facts at the olog level

Another fact one might wish to record is that “John Doe’s weight is 150 lbs.” This is established by declaring that the following diagram commutes:
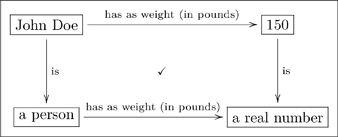
(18)


If one only had the top line, it would be less obvious how to connect its information with that of other ologs. (See Section 4 for more on connecting different ologs).

Note that the top line in Diagram (18) might also be considered as existing at the “data level” rather than at the “olog level.” In other words, one could see John Doe as an “instance” of 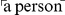, rather than as a type in and of itself, and similarly see 150 as an instance of 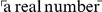. This idea of an olog having a “data level” is the subject of the Section 3.

#### 2.3.4 Rules of good practice

A fact is the declaration that two paths (having the same source and target) in an olog are equivalent. Such a fact is either presented as a checkmark between the two paths (if such a check-mark is unambiguous) or by an equation. Every such equivalence should be declared; i.e., no fact should be considered too obvious to declare.

## 3 Instances

The reader at this point hopefully sees an olog as a kind of “concept map,” and it is one, albeit a concept map with a formal structure (implicitly coming from category theory) and specific rules of good practice. In this section we will show that one can also load an olog with data. Each type can be assigned a set of instances, each aspect will map the instances of one type to instances of the other, and each fact will equate two such mappings. We give examples of these ideas in Section 3.1.

In Section 3.2, we will show that in fact every olog can also serve as the layout for a database. In other words, given an olog one can immediately generate a *database schema*, i.e., a system of tables, in any reasonable data definition language such as that of SQL. The tables in this database will be in one-to-one correspondence with the types in the olog. The columns of a given table will be the aspects of the corresponding type, i.e., the arrows whose source is that type. Commutative diagrams in the olog will give constraints on the data.

In fact, this idea is the basic thesis in FDM, even though the word olog does not appear in that paper. There it was explained that a category 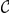 naturally can be viewed as a database schema and that a functor 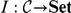, where 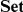 is the category of sets, is a database state. Since an olog is a drawing of a category, it is also a drawing of a database schema. The current section is about the “states” of an olog, i.e., the kinds of data that can be captured by it.

### 3.1 Instances of types, aspects, and facts

Recall from Section 2 that basic ologs consist of types, displayed as boxes; aspects, displayed as arrows; and facts, displayed as equations or check-marks. In this section we discuss the instances of these three basic constructions. The rules of good practice (2.1.1, 2.2.1, and 2.3.4) were specifically designed to simplify the process of finding instances.

#### 3.1.1 Instances of types

According to Rules 2.1.1, each box in an olog contains text which should refer to **a distinction made and recognizable by the author for which instances can be documented.** For example if my olog contains a box
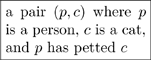
(19)


then I must have some concept of when this situation occurs. Every time I witness a new person-cat petting, I document it. Whether this is done in my mind, in a ledger notebook, or on a computer does not matter; however using a computer would probably be the most self-explanatory. Imagine a computer program in which one can create ologs. Clicking a text box in an olog results in it “opening up” to show a list of documented instances of that type. If one is reading the CBS news olog and clicks on the box 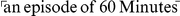, he or she should see a list of all episodes of the TV show “60 Minutes.” If we wish to document a new person-cat petting incident we click on the box in (19) and add this new instance.

#### 3.1.2 Instances of aspects

According to Rules 2.2.1, each arrow in an olog should be labeled with text that refers to a functional relationship between the source box and the target box. A functional relationship 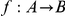 between finite sets 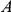 and 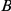 can always be written as a 2-column table: the first column is filled with the instances of type 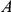 and the second column is filled with their 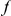-values, which are instances of type 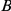.

For example, consider the aspect 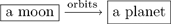
(20)


We can document some instances of this relationship using the following table
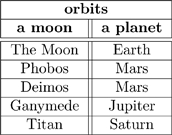
(21)


Clearly, this table of instances can be updated as more moons are discovered by the author (be it by telescope, conversation, or research).

The correspondence between the aspect in (20) and Table (21) makes it clear that ologs can serve to hold data which exemplifies the author’s world-view. In Section 3.2, we will show that ologs (which have many aspects and facts) can serve as bona fide database schemas.

#### 3.1.3 Instances of facts

Recall the following olog:
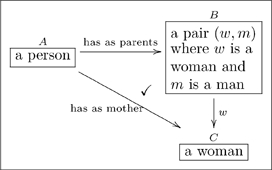
(15)and consider the following instances of the three aspects in it
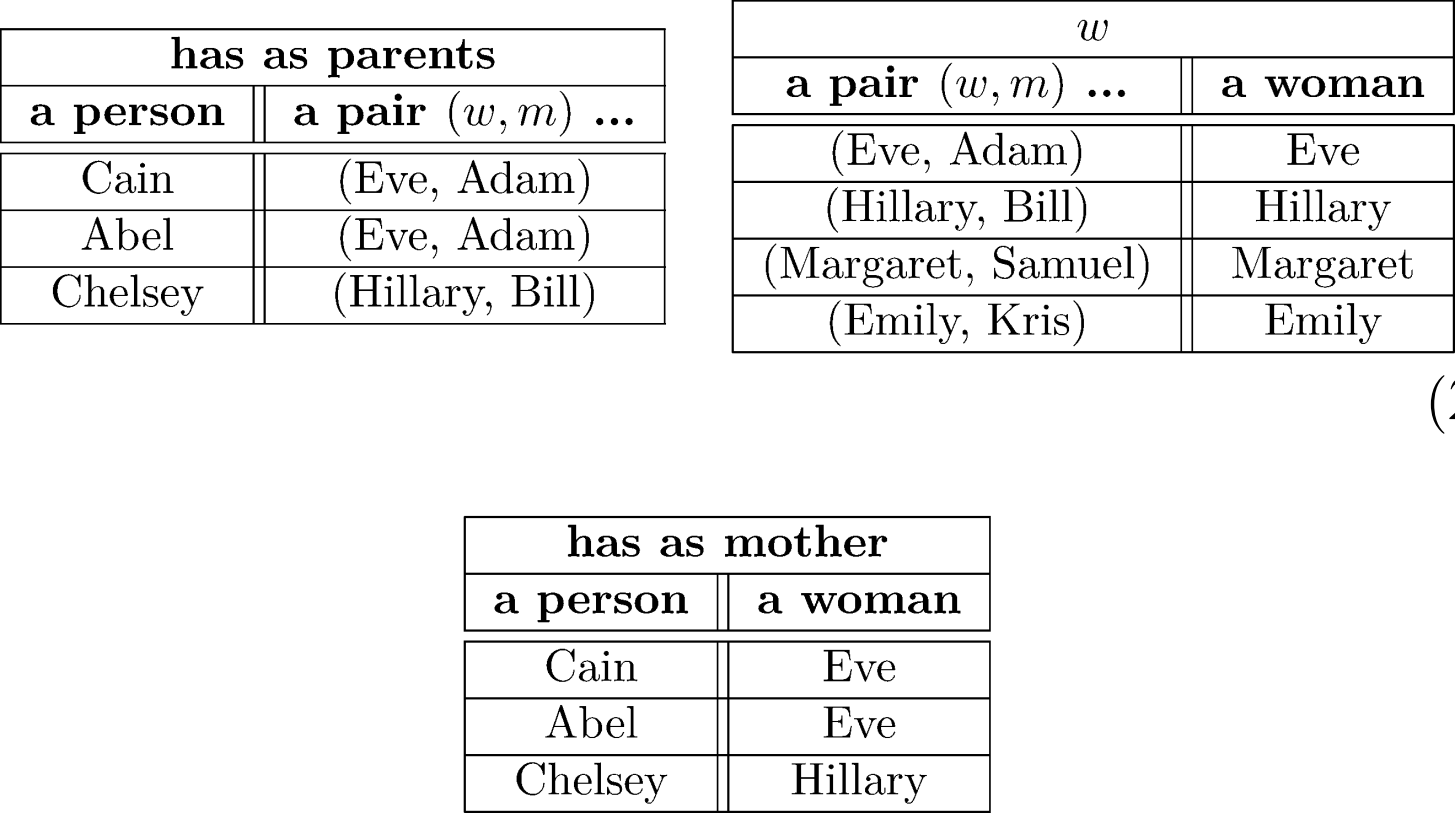
(22)


When we declare that the diagram in (15) commutes (using the check-mark), we are saying that for every instance of 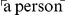 (of which we have three: Cain, Abel, and Chelsey), the two paths to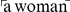 give the same answers. Indeed, for Cain the two paths are:

i. Cain 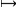 (Eve, Adam) 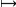 Eve;

ii. Cain 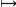 Eve;

and these answers agree. If one changed any instance of the word “Eve” to the word “Steve” in one of the tables in (22), some pair of paths would fail to agree. Thus the “fact” that the diagram in (15) commutes ensures that there is some internal consistency between the meaning of parents and the meaning of mother, and this consistency must be born out at the instance level.

All of this will be formalized in Section 3.2.2.

### 3.2 The relationship between ologs and databases

Recall from Section 3.1.1 that we can imagine creating an olog on a computer. The user creates boxes, arrows, and compositions, hence creating a category 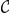. Each text-box 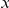 in the olog can be “clicked” by the computer mouse, an action which allows the user to “view the contents” of 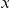. The result will be a set of things, which we might call 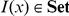, whose elements are things of type 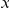. So clicking on the box 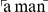 one sees 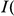 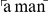 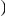, the set of everything the author has documented as being a man. For each aspect 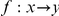 of 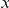, the user can see a function from the set 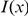 to 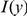, perhaps as a 2-column table as in (22).

The type 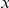 may have many aspects, which we can put together into a single multi-column table. Its columns are the aspects of 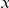, and its rows are the elements of 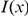. Consider the following olog, taken from FDM where it was presented as a database schema.
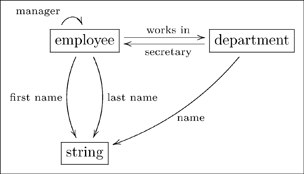
(23)


The type 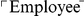 has four aspects, namely manager (valued in 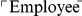), works in (valued in 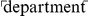), and first name and last name (valued in 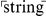). As a database, each type together with its aspects form a multi-column table, as in the following example.

#### 3.2.1 Example

We can convert Olog (23) into a database schema. Each box represents a table, each arrow out of a box represents a column of that table. Here is an example state of that database.
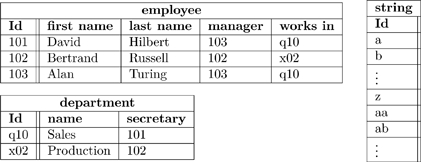
(24)


Note that every arrow 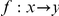 of Olog (23) is represented in Database (24) as a column of table 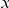, and that every cell in that column can be found in the Id column of table 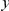. For example, every cell in the “works in” column of table **employee** can be found in the Id column of table **department**.

The point is that ologs can be drawn to represent a world-view (as in Section 2), but they can also store data. Rules 1,2, and 3 in 2.1.1 align the construction of an olog with the ability to document instances for each of its types.

#### 3.2.2 Instance data as a set-valued functor

Let 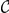 be an olog. Section 3 so far has described instances of types, aspects, and facts and how all of these come together into a set of interconnected tables. The assignment of a set of instances to each type and a function to each aspect in 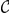, such that the declared facts hold, is called an assignment of *instance data* for 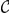. More precisely, instance data on 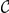 is a functor 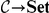, as in Definition 3.2.3.

#### 3.2.3 Definition

Let 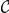 be a category (olog) with underlying graph 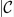, and let 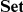 denote the category of sets. An *instance of* 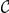 (or *an assignment of instance data for* 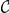) is a functor 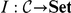. That is, it consists of

• a set 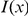 for each object (type) 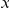 in 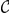,

• a function 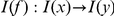 for each arrow (aspect) 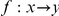 in 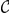, and

• for each fact (path-equivalence or equation)
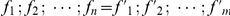



declared in 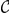, an equality of functions
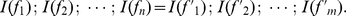



The symbol ‘
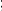
’ in paths denotes concatenation or formal composition. If we let 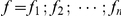 and 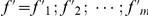 denote two paths, then we often write 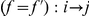 to denote the fact that these paths are equivalent.

## 4 Communication between ologs

The world is inherently heterogeneous. Different individuals in the world naturally have different world-views – each individual has its own perspective on the world. By an individual we mean either an individual person acting on their own, a community acting as a single entity, a software agent, etc. Later in this section we will use the notion of a community acting as a distributed collection of linked, yet independent, individuals. The conceptual knowledge (information resources) of an individual represents its world-view, and is encoded in an ontology log, or olog, containing the concepts, relations, and observations that are important to that individual. An olog is a formal specification of an individual’s world-view in a language representing the concepts and relationships used by that individual. In addition to the formulation of an expressive language, a specification needs to contain axioms (facts) that constrain the possible interpretations of that language.

Since the ologs of different individuals are encoded in different languages, the important need to merge disparate ologs into a more general representation is difficult, time-consuming and expensive. The solution is to develop appropriate communication between individuals to allow interoperability of their ologs. Communication can occur between individuals when there is some commonality between their world-views. It is this commonality that allows one individual to benefit from the knowledge and experience of another. In this section we will discuss how to formulate these channels of communication, thereby describing a generalized and practical technique for merging ologs.

The mathematical concept that makes it all work is that of a functor. A functor is a mapping from one category to another that preserves all the declared structure. Whereas in Definition 3.2.3 we defined a functor from an olog to 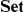, here we will be discussing functors from one olog to another.

Suppose we have two ologs, 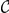 and 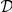, that represent the world-views of two individuals. A functor 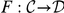 is basically a way of matching each type (box) of 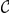 to a type of 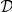, and each aspect (arrow) in 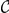 to an aspect (or path of aspects) in 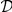. Once ologs are aligned in this way, communication can occur: the two individuals know what each other is talking about. In fact, mathematically we can show that instance data held in 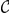 can be transformed (in coherent ways) to instance data held in 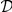, and vice versa (see FDM). In simple terms, once individuals understand each other in a certain domain (be it social, mathematical, etc.), they can communicate their views about it.

While the basic idea is not hard, the details can be a bit technical. This section is written in a more formal and logical style, and is decidedly more difficult than the others. For this section only, we assume the reader is familiar with the notion of fibered categories, colimits in the category 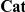 of categories, etc. We return to our more informal style in Section 5, where we discuss how an individual can author a more expressive olog.

### 4.1 Categories and their presentations

We never defined categories in this paper, but we defined ologs and said that the two notions amounted to the same thing. Thus, we implied that a category consists of the following: a set of objects, a set of arrows (each pointing from one object to another), and a congruence relation on paths; a congruence relation on paths is an equivalence relation on paths that respects endpoints and is closed under composition from left and right (see the axioms in 25). This differs from the standard definition of categories (see [6]), which replaces our congruence relation with a composition rule and associativity law (obtained by taking the categorical quotient). One could say that an olog is a presentation of a category by generators (objects and arrows) and relations (path congruences). Any category can be resolved and presented in such a way, which we will call a specification. Likewise any functor can be resolved and presented as a morphism between specifications. We take an agnostic approach to foundations here. With the presentation form, we show how categories and functors are definable in terms of sets and functions, indicating how category theoretic concepts could be defined in terms of set theory. However, we fully understand that 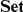, the category of sets and functions, is but one example of a topos, indicating how set theoretic concepts could be defined in terms of category theory.

In fact, this presentation form for categories (and the analogous one for functors) is preferable for our work on communication between ologs, because it separates the strictly graphical part of an olog (its types and aspects, regarded as the olog language) from the propositional part (its facts, regarded as the olog formalism). This presentation form is standard in the institutions [13] and information flow [14] communities, since it separates the mechanism of flow from the content of flow; in this case the formal content. Our work here applies the general theories of institutions and information flow to the sketch logical system Sk (in its various manifestations) that underlies categories and functors, demonstrating how this logical system can be used for knowledge representation. Using the presentation forms for categories and functors, we show how communication between individuals is effected by the flow of information along channels.

### 4.2 The architecture underlying information systems

We think of a community of people, businesses, etc. in terms of the ologs of each individual participant together with the information channels that connect them. These channels are functors between ologs, which allow communication to occur. The heterogeneity of multiple differing world-views connected through such links can lead to a flexibility and robustness of interaction. For example, heterogeneity allows for multiple schemas to be employed in the design of database systems in particular, and multiple languages to be employed in the design of knowledge representation systems in general.

For any olog, consider the underlying graph of types and aspects. We regard this graph as being the language of the olog, with the facts of the olog being a subset of all the possible assertions that one can make within this language (in the other direction, Section 4.4 indicates how natural languages can be encoded into ologs). Any two ologs with the same underlying graph of types and aspects have the same language, and since the facts of each olog are expressed in the same language, they can be “understood” by each other without translation. As such, we think of the collection of all ologs with the same language (underlying graph) as forming a homogeneous *context*, with the ologs ordered in a specialization-generalization hierarchy.

Whereas an olog represents (the world-view of) a single individual, an information system (of ologs) represents a community of separate, independent and distributed individuals. Here we consider an information system to be a diagram of ologs of some shape 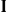; that is, a collection of ologs and constraints indexed by a base category 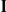. The parts of the system represent either the ologs of the various individuals in the system or common grounds needed for communication between the individuals. Each part of the system specifies its world-view as facts expressed in terms of its language. The system is heterogeneous, since each part has a separate language for the expression of its world-view. The morphisms between the parts are the alignment (constraint) links defining the common grounds.

As will be made clear in a moment, there is an underlying distributed system consisting of the language (underlying graph) for each component part of the information system and a translation (graph morphism) for each alignment link. We can think of this distributed system as an underlying system of languages linked by translating dictionaries. This distributed system determines an information channel with core language (graph) and component translation links (graph morphisms) along which the specifications of each component part can flow to the core. We can think of this core as a universal language for the whole system and the channel as a translation mechanism from parts to whole. At the core, the direct flow of the component specifications are joined together (unioned) and allowed to interact through entailment. The result of this interaction can then be distributed back to the component parts, thereby allowing the separate parts of an information system to interoperate.

In this section, we will make all this clear and rigorous. As mentioned above, we will work with category presentations (here called *specifications*) rather than categories. We will discuss the homogeneous contexts called fibers in detail and give the axioms of satisfaction. We will then discuss how morphisms between graphs (the translating dictionaries between the ologs) allow for direct and inverse information flow between these homogeneous fiber contexts. Finally, we discuss specifications (also known as *theories*) and the lattice of theories construction for ontologies.

In Section 4.3 we will discuss how the information in ologs can be aligned by the use of common grounds. This alignment will result in the creation of *information systems*, which are systems of ologs connected together along functors. We will discuss how to take the information contained in each olog of a heterogeneous system and integrate it all into a single whole, called the fusion olog. Finally we will discuss how the consequence of bringing all this information together, and allowing it to interact, can be transferred back to each part of the system (individual olog) as a set of local facts entailed by remote ologs, allowing for a kind of interoperability between ologs. In Section 4.4 we will discuss conceptual graphs and their relationship to ologs.

#### 4.2.1 Fibers

A graph 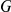 contains types as nodes and aspects as edges. The graphs underlying an olog is considered its *language*. Any category 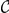 has an underlying graph 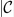. In particular, 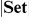 is the graph underlying the category of sets and functions. Olog (12) has an underlying graph containing the three types 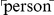, 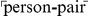 and 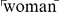 and the three aspects ‘has a parent’, ‘woman’ and ‘has as mother’. Olog (17) has an underlying graph containing the three types 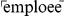,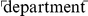, and 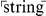 and the six aspects ‘manager’, ‘works in’, ‘secretary’, ‘name’, ‘first name’ and ‘last name’. Let 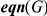 denote the set of all facts (equations) that are possible to express using the types and aspects of 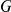. A 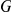-specification is a set 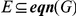 consisting of some of the facts expressible in 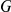. The singleton set with the one fact that “the female parent of a person is his/her mother” is a specification for the graph of Olog (12). The set with the two facts that “the manager has the same department as any employee” and “the secretary of a department is an employee in that department” is a specification for the graph of Olog (17). Let 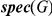 denote the collection of all 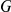-specifications ordered by inclusion 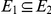.

#### 4.2.2 Satisfaction

It will be useful here to define an instance of a graph 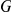, instead of an instance of a category 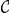. An instance of a graph populates the graph by assigning instance data to it. An instance of a graph 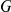 is a graph morphism 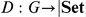 mapping each type 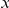 in 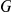 to a set 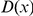 of instances and mapping each aspect 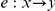 in 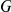 to an instance function 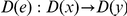. Using database terminology, we also call 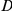 a key diagram, since it gives the set of row identifiers (primary keys) of tables and the cell contents defined by key maps.

A key diagram 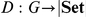 satisfies (is a model of) a 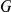-fact 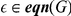 (see Definition 3.2.3), symbolized 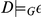, when we have an equality of functions 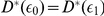. We also say that 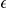 (holds in) is true when interpreted in 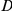. An identity 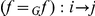 holds in all key diagrams (hence, is a tautology), and vice-versa for any set 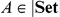 a constant key diagram 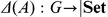 satisfies any fact 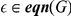. A key diagram 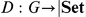 satisfies (is a model of) a 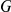-specification 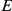, symbolized 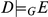, when it satisfies every fact in the specification. For any graph 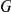, a 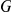-specification 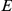 entails a 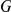-fact 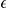, denoted by 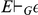, when any model of the specification satisfies the fact. The consequence 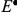 of a 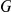-specification 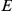 is the set of all entailed equations. The consequence operator 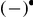 is a closure operator, and the consequence of a specification is a congruence. For any 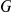-specification 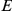, entailment satisfies the follow axioms.
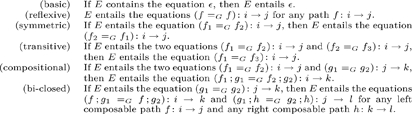
(25)


These are converted to inference rules in Table 1. To construct 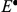, we first take the reflexive, symmetric, and transitive closure 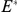 of 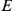 (so that 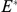 is a 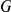-specification and also the smallest equivalence relation containing 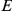), and then we get 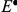 by closing up under composition on left and right. We extend specification inclusion with the entailment order, where 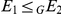 when 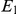 entails each equation in 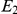; that is, when 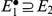 or equivalently when 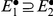. The statement “
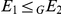
” asserts that 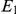 is at least as specialized as 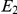. The entailment order 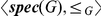, which is a specialization-generalization order, represents a local version of the “lattice of theories” construction of Sowa [15] (see Section 4.2.5). The opposite entailment order 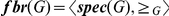 is called the fiber order. For consistency in discussion, we follow the terminology of formal concept analysis [16], information flow [14] and the theory of institutions [13]. This includes the polarity induced by concept lattices and the directionality of infomorphisms. In the lattice 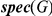 (this is a complete preorder, loosely called a “lattice”), the meet is union 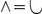 and the join is intersection 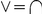; whereas in the lattice 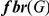, the join is union 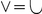 and the meet is intersection 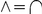. Any specification 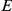 is entailment equivalent to its consequence 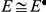. A specification 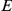 is closed when it is equal to its consequence 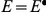. There is a one-one correspondence between closed 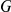-specifications and categories over graph 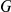. The conceptual intent of a key diagram 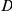, implicit in satisfaction, is the closed specification 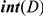 consisting of all facts satisfied by the key diagram. Hence, 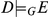 iff 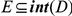 iff 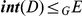. This equivalence between satisfaction and entailment order is the first step in the algebraization of Tarski’s “semantic definition of truth”.

**Table 1:** Inference Rules.

**equivalence:**	(reflexive)	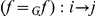
	(symmetric)	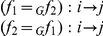
	(transitive)	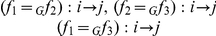
**algebra:**	(compositional)	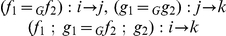
	(bi-closed)	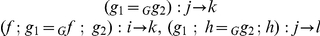
**morphic flow:**	(direct)	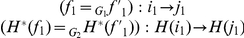
	(inverse)	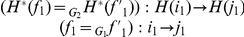
**system flow:**	(direct)	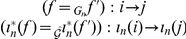
	(inverse)	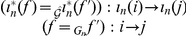

#### 4.2.3 Elementary flow

A graph morphism 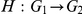 maps the types and aspects of 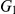 to the types and aspects of 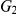. Graph morphisms are the translations between ologs. A functor 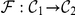 has an underlying graph morphism 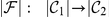. For any graph morphism 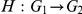, there is a fact function 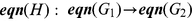 that maps a 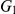-equation 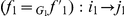 to the 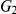-equation 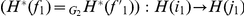, and a key diagram functor 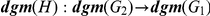 that maps a key diagram 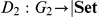 to the key diagram 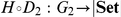 (the composition of graph morphisms is written in diagrammatic order). At the abstraction of institutions [17], the fact function is the fundamental unit of information (formal) flow for ologs, and the key diagram functor is the fundamental unit of semantic flow for ologs. Formal flow is adjoint to semantic flow – satisfaction is invariant under flow: 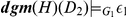 iff 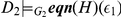 for any graph morphism 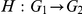, source fact 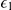 and target diagram 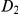. Specifications can be moved along graph morphisms by extending the fact (equation) function. For any graph morphism 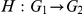, define the *direct flow* operator 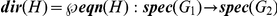 to be the direct image function, and the *inverse flow* operator 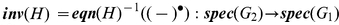 to be the composition of the specification consequence operator followed by the inverse image function. Direct and inverse flow are adjoint monotonic functions 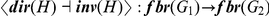 w.r.t. fiber order: 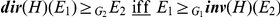. For any graph morphism 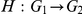, any 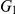-specification 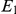, and any 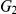-specification 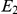, entailment satisfies the following axioms.
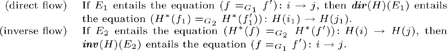



These are converted to inference rules in Table 1. A graph morphism 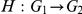 defines a consequence operator 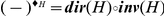 on the fiber preorder 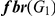, where 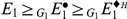.

#### 4.2.4 Specifications

A specification 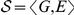 is an indexed notion consisting of a graph 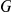 and a 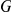-specification 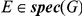. It is sometimes convenient to use the symbol ‘
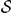
’ in place of ‘
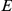
’; for example, to say that “
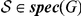
”. A category 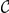 can be resolved and presented as a specification 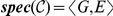 consisting of the underlying graph 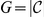 containing the types and aspects of 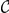 and the collection 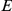 of all facts that hold in 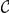. In the other direction, any specification 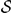 induces a (quotient) category 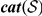. Olog (12) and Olog (17) are described as specifications in Section 4.2.1. A specification morphism 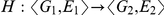 is a graph morphism 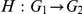 that preserves entailment: 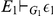 implies 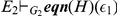 for any 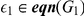; or equivalently that satisfies the adjointness conditions, 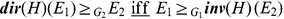. Being a graph morphism, it maps types to types and aspects to aspects. Moreover, it also maps facts in 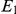 to facts in 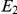; that is, it preserves all the declared structure. A functor 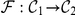 can be resolved and presented as a specification morphism 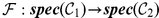. Hence, the presentation form for a functor does exactly what the functor does. The fibered category of specifications 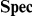 has specifications as objects and specification morphisms as morphisms. Thus, it is defined in terms of information flow. There is an underlying graph functor 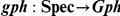 from specifications to graphs 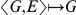. The subcategory over any fixed graph 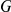 is the fiber 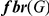; because of the opposite orientation, we say that “the category of specifications points downward in the concept lattice”. Throughout this section we identify ologs with specifications and olog morphisms with specification morphisms.

#### 4.2.5 The lattice of theories construction

Sowa’s “lattice of theories” construction (LOT) describes a modular framework for ontologies [15]. The Olog formalism follows the approach to LOT described in [18], where the IFF term ‘theory’ is replaced by the Olog term ‘specification’ or ‘olog’. In the Olog formalism, LOT is locally represented by the entailment preorders 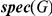, and globally represented by the category of specifications 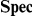. We follow the discussion in Section 6.5 “Theories, Models and the World” of Sowa [15]. From each olog (specification) in the “lattice of theories”, the entailment ordering defines paths to the more generalized ologs above and the more specialized ologs below. Sowa defines four ways for moving along paths from one olog to another: contraction, expansion, revision and analogy.

**Contraction:** Any olog can be contracted or reduced to a smaller, simpler olog, moving upward in the preorder 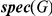, by deleting one or more facts.

**Expansion:** Any olog can be expanded, moving downward in the preorder 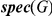, by adding one or more facts.

**Revision:** A revision step is composite, moving crosswise in the preorder 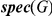; it uses a contraction step to discard irrelevant details, followed by an expansion step to added new facts.

**Analogy:** Unlike contraction and expansion, which move to nearby ologs in an entailment preorder 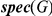, analogy moves to an olog in a remote entailment preorder in the category 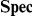 via the flow along an underlying graph morphism 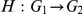 by systematically renaming the types and aspects that appear in the facts: any olog 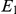 in 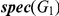 is moved (by systematic renaming) to the olog 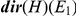 in 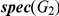.

According to Sowa, the various methods used in nonmonotonic logic and the operators for belief revision correspond to movement through the lattice of theories.

### 4.3 Alignment and integration of information systems

#### 4.3.1 Common ground

Roughly speaking, an olog morphism 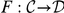 is *meaningful* when for each type 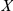 in 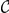, every intended instance of 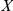 in 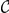 would be considered an instance of 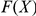 by the author of 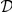 (in which case we say the intention for types is respected), and in a similar way the intention for aspects is respected. Precisely speaking, if 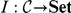 and 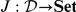 are instance data for 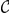 and 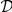, then 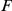 is meaningful relative to 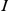 and 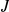 if one can exhibit a natural transformation 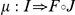 as in FDM.

Given the world-views of two individuals, as represented by ologs 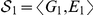 and 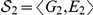, there is little hope that one of them completely contains the other (even after allowing for renaming of types and aspects), and there is correspondingly little chance of finding a meaningful olog morphism between the two. Instead, in order to communicate the two individuals could attempt to find a common ground, a third olog 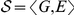 and meaningful morphisms 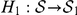 and 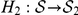 (a common ground olog is also called a reference ontology in knowledge representation). This connection is a 1-dimensional knowledge network 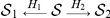 of shape 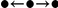 called a span (in 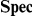), where each node is an olog and each edge is a morphism between ologs. The requirements of this span are that 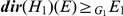 and 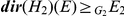, two requirements involving local flow. Equivalently, that 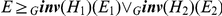. The latter precise expression can be rendered in natural language as “the world-view of the common ground is contained in the combined world-views of the two individuals”. The various local direct/inverse flows allow world-views to be compared. Such a common ground can be expanded and improved over time. The basic idea is that one individual can attempt to explain a new idea (type, aspect or fact) to another in terms of the common ground. Then the other individual can either interpret this idea as they already have, learn from it (i.e., freely add it to their olog), or reject it. At the abstraction of institutions [17], an olog morphism 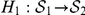 is an atomic constraint (alignment) link between 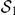 and 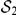. Following this, we view a common ground span 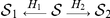 as a molecular constraint between 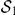 and 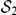, which is weakest when 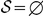 and strongest when 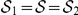.

#### 4.3.2 Systems of ologs

In the general case, more than two individuals will share a common ground. For example, companies that do business together may have a common-ground olog as part of a legal contract; or, the various participants at a conference will have some common understanding of the topic of that conference. In fact, for any finite set of ologs 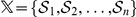, there should be a common ground world-view (even if empty), say 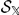. If 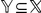 is a subset, then there should be a map 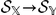 because any common understanding held by the individuals in 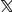 is held by the individuals in 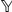. For example, the triangular-shaped diagram
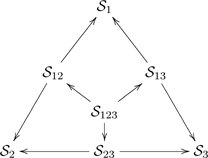
(26)


represents three individuals 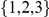, their ologs 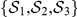, their pair-wise common ground ologs 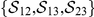, and their three-way commonality olog 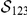. This diagram, which stands for the interaction between individuals 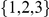, does not stand alone, but is part of an intricate web of other ologs and alignment constraints. In particular, individuals 1 and 3 may be part of some different interacting group, say of individuals 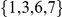, and hence the right edge of the diagram would be part of some tetrahedron-shaped diagram with vertices 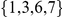. If we take the point-of-view that “a collection of ologs representing the world-views of various individuals” is a system, then we can think of the ologs as being the types of that system, the morphisms connecting the ologs as being the aspects of that system, with the shape of a system being its underlying graph. In essence, we can apply ologs to themselves. In the system represented by diagram (26), there are seven types 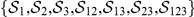 and nine aspects 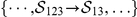, and the shape is the graph in the diagram (27).
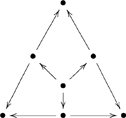
(27)


In addition, we can introduce certain facts to represent the meaning of that system and then enforce those facts.

A *distributed system* is a diagram (functor) 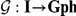 of shape 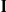 within the ambient category 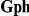. As such, it consists of an indexed family 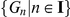 of graphs together with an indexed family 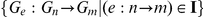 of graph morphisms. Let 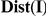 denote the collection of distributed systems of shape 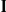. An *information system* is a diagram 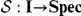 of shape 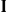 within the ambient category 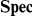. As such, it consists of an indexed family 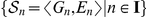 of ologs together with an indexed family 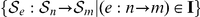 of olog morphisms. Some of these ologs might represent the world-views of various individuals, whereas others could be common grounds; also included might be portals between individual ologs and common grounds, as in the CG example of Section 4.4. Let 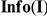 denote the collection of information systems of shape 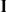. An information system 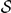 with component ologs 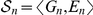 has an underlying distributed system 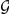 of the same shape with component graphs 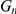 for 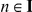. For any distributed system 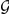, let 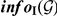 denote the collection of information systems over 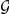 of shape 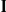. There is a pointwise entailment order 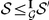 on 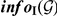 when component ologs satisfy the same entailment ordering 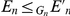 for 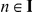, and by taking the coproduct there is a pointwise entailment order on 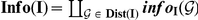. A constant distributed system 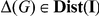 is a distributed system 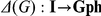 with the same language 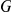 for any index 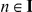. Any constant distributed system defines join and meet monotonic functions 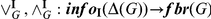 mapping an information system 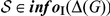 to the join and meet ologs 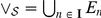 and 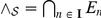 in 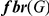. The join monotonic function is adjoint to the constant monotonic function 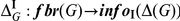 that distributes an olog 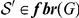 to the various locations 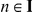 forming a constant information system 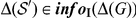, since 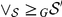 iff 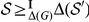 for any system 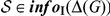 and any olog 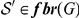.

#### 4.3.3 System morphisms

Just as ologs are linked by morphisms, information systems are also linked by morphisms. For these there is the new complication of shape. In this paper we define fixed-shape system moorphisms, but a more general definition would allow the shape to vary. A distributed system morphism 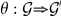 in 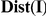 consists of a collection 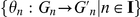 of component graph morphisms, which are systematically coordinated in the sense that they satisfy the naturality conditions 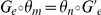 for any indexing link 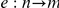 in 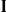. A direct flow operator 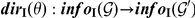 along 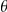 can be define, which maps an information system 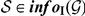 to an information system 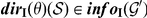 defined by 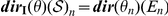 for 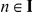. This is well-defined, since 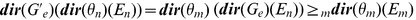. An inverse flow operator 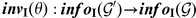 can similarly be defined. Direct and inverse flow are adjoint monotonic functions 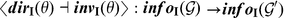, since 
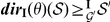
 iff 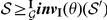. An information system morphism 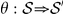 in 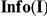 consists of a collection 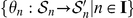 of component olog morphisms, which are systematically coordinated and preserve alignment in the sense that they satisfy the naturality conditions 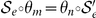 for any indexing link 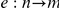 in 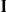; equivalently, 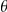 is a morphism between the underlying distributed systems 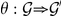 and the direct flow of 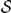 is at least as general as 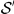: 
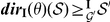
. The ordering 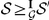 is an information system morphism 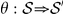 with identity component translations 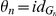 for each index 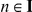.

#### 4.3.4 Channels

We continue with our systems point-of-view. Since we have represented the whole system as a diagram 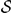 of parts (ologs) 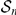 with part-part relations (alignment constraints) 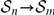, we also want to represent the whole system as an olog 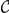 with part-whole relations 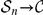. The theory of part-whole relations is called mereology. It studies how parts are related to wholes, and how parts are related to other parts within a whole. An *information channel* 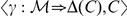 consists of an indexed family 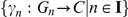 of graph morphisms called flow links with a common target graph 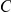 called the core of the channel. A channel 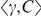 covers a distributed system 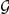 of shape 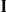 when the part-whole relationships respect the alignment constraints (are consistent with the part-part relationships): 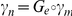 for each indexing morphism 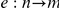 in 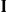. A covering channel is a distributed system morphism 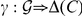 in 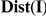 from distributed system 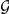 to constant distributed system 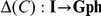. Such a channel defines a direct flow operator 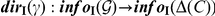 and an inverse flow operator 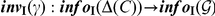. For any two covering channels 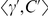 and 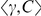 over the same distributed system 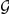, a refinement 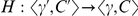 is a graph morphism between cores 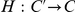 that respects the part-whole relationships of the two channels: 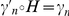 for 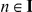. In such a situation, we say the channel 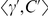 is a refinement of the channel 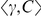. A channel 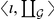 is called a minimal cover (using information flow terminology [14]) or an optimal(ly refined covering) channel of a distributed system 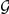 when it covers 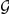 and for any other covering channel 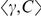 there is a unique refinement 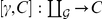 from 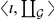 to 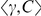.

#### 4.3.5 System flow

In order to represent an information system 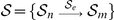 as a single olog 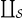, called the fusion of 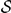, with part-whole relations 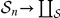, we follow the colimit theorem of [19] by recognizing the following three properties.

• Optimal channels exist for any distributed system 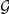.

• 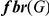 is a complete preorder for any graph 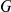, loosely called a “lattice”.

• For any graph morphism 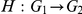, direct and inverse flow are adjoint monotonic functions 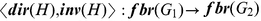.

Let 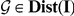 be a distributed system of shape 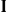 with optimal channel 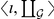. The optimal core 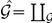 is called the sum of the distributed system 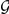, and the optimal channel components (graph morphisms) 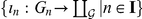 are called flow links. There is a *direct system flow* monotonic function (see Diagram 28) 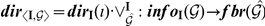. Direct system flow has two steps: (i) direct (fixed shape) system flow of an information system along the optimal channel (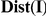-morphism) 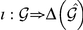 and (ii) lattice join combining the contributions of the parts into a whole. In the opposite direction, there is an *inverse system flow* monotonic function (see Diagram 28) 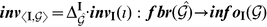. Inverse system flow has two steps: (i) mapping an olog with core language 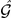 to a constant information system over 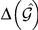 with shape 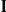 by distributing the olog to the locations 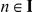, and (ii) inverse (fixed shape) system flow of this constant information system back along the optimal channel 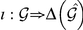. Direct system flow is adjoint to inverse system flow 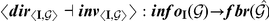, since the composition components are adjoint. For any distributed system 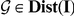 with optimal core 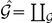, any information system 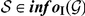, and any olog 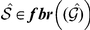, entailment satisfies the following axioms.
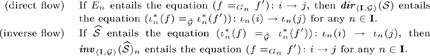



These are converted to inference rules in Table 1.
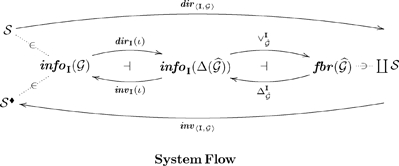
(28)


Information flow can be used to compute the fusion olog for an information system and to define the consequence of an information system. Fusion is direct system flow, and consequence is the composition of direct and inverse system flow. Let 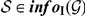 be any information system. The fusion 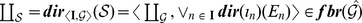 is an olog that represents the whole system in a centralized fashion [20],[17]. The consequence 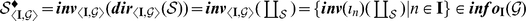 is an information system that represents the whole system in a distributed fashion [17]. It is inverse flow of the fusion olog along the optimal channel, transfering the entailed facts of the whole system to the component parts. By allowing system shape to vary, channels can be generalized to morphisms of distributed systems. Then a notion of relative fusion (direct system flow) can be defined in terms of left Kan extension, and a notion of relative system consequence can be defined as the composition of direct followed by inverse system flow.

The consequence operator 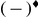, which is defined on information systems, is a closure operator on the complete preorder 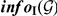, and by taking the coproduct it is a closure operator on the complete preorder 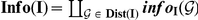: (increasing) 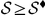, (monotonic) 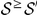 implies 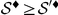 and (idempotent) 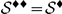. Pointwise entailment order 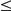 on 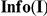 is only a preliminary order, since it does not incorporate interactions between system component parts. System entailment order 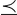 on 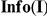 is defined by 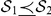 when 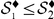; equivalently, 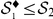. Pointwise order is stronger than system entailment order: 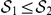 implies 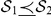. This is a specialization-generalization order. Any information system 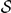 is entailment equivalent to its consequence 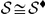. An information system 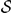 is closed when it is equal to its consequence 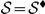.

The whole effect of taking the system consequence may be greater than the sum of its parts, in the sense that 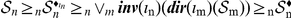 for any 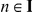, since separate parts may have a productive interaction at the channel core. A final part of an information system is a part with no non-trivial constraint links from it. (The graphical subsystem beneath) nonfinal parts are necessary for the alignment of information systems, resulting in the equivalencing of types and aspects through quotienting. However, because of the covering condition 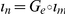 and the entailment order 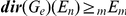 for constraint links 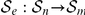, only the fact(ual) content of final parts of information systems are necessary to compute the system fusion and consequence.

#### 4.3.6 General examples

Here are some examples of system fusion/consequence.

• An information system 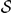 with a constant underlying distributed system, 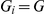 for all 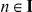, gathers together all the component parts of the information system and forms their consequence. It has identity flow links 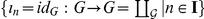, compo nent join fusion 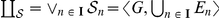, and constant system consequence 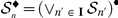 for all 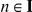.

• A discrete information system 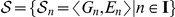 with no constraint links 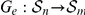 for 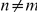, has coproduct injection flow links 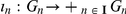, non-restricting fusion, and inverse flow projecting back to individual component consequence 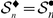 for all 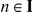. No alignment (constraint) links means no interaction.

• An information system 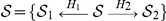 consisting of a single common ground 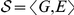 between two component ologs 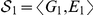 and 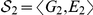, with underlying distributed system (span) 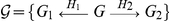, has pushout injection flow links 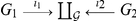, direct image union fusion 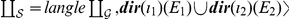, and system consequence components 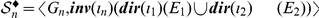 for 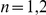. The flow links will quotient any types and aspects that are connected through the common ground allowing for the approprate interaction in the fusion conse quence 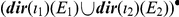, then the inverse flow will reconnect this with the component types and aspects.

### 4.4 Conceptual graphs

The conceptual graph formalism (CG) for knowledge representation [15], was initially formulated to represent database systems (DBS), but is now used in natural language processing (NLP) and first-order logic (FOL). Verbs in NLP can often be represented relationally by star(-shaped conceptual) graphs. For example, the sentence “John is going to Boston by bus” might be represented by the conceptual graph
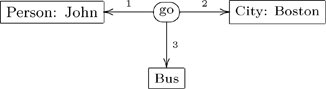
(29)


In a sentence of natural language, thematic roles are semantic descriptions of the way (the entities described by) a noun phrase functions with respect to (the action of) the verb. These entities are the participants in the occurrent expressed by the verb. For the action of ‘going’ in the above sentence there are three participants and hence three thematic roles. ‘John’ plays the role of the agent of the action, a ‘Bus’ is the instrument used in the action and ‘Boston’ is the destination of the action. Translations using thematic roles can be used to align two ontologies with respect to a common ground. A CG-style translation of conceptual graph (29) would replace the verb relation ‘going’ with a concept ‘Go’ and replace the edges that form the signature of the ‘going’ relation with binary relations for the three roles ‘agent’, ‘instrument’ and ‘destination’.
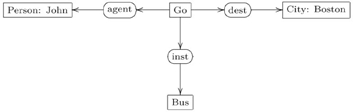
(30)


However, the case relations that semantically describe the thematic roles should be viewed as functional in nature; that is, for any instance of the action of a sentence’s verb there is a unique entity described by a noun phrase of the sentence. When this semantics is respected, the translation to thematic roles becomes a process of “linearization”, which is best described abstractly as: (1) the identification of relation types with entity types, (2) the translation of a sorted multiarity relation to a span of functions, one function for each role, and (3) the functional interpretation of thematic roles.

The Olog formalism, which also represents DBS and NLP, is a version of equational logic. Both the Olog and CG formalisms were designed as graphical representations. However, the CG formalism is binary and relational, whereas the Olog formalism is unary and functional. The CG formalism is binary since it has two kinds of type, concepts and relations; it is relational in the way it interprets edges. The Olog formalism is unary since it has only one kind of type, the abstract concept; it is functional in the way it interprets aspects (edges). However, much of the semantics of the CG formalism can be transformed to the Olog formalism by the process of linearization, thereby gaining in efficiency and conciseness. This linearization process works for any binary/relational knowledge representation, such as CGs, entity-relationship data modelling [21], relational database systems or the Information Flow Framework [22]. In the entity-relationship data modelling, 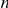-ary relationship links are replaced by 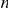-ary spans of aspects and attributes are included as types.
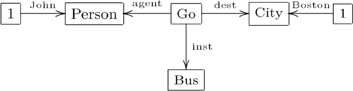
(31)


For example, the conceptual graph (29) can be linearized to the olog graph in diagram (31), where 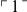 is the universal type to which all types have a unique aspect. Since olog aspects are interpreted functionally, the functional nature of thematic roles is respected. In this manner, the olog formalism could be used to replace the CG representation of ontologies. For example, a community (acting as an individual) could build its ontology 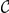 from ground up by aligning it with some top-level reference ontology 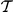 (such as in the appendix of [15]), thereby importing some formal semantics from 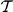. The following fragment demonstrates how this works.

Assume that ontology 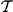 contains the concept of “spatial process” as represented by the general concept type with aspects 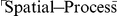 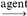 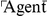, 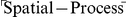 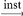 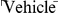 and 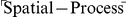 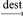 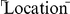. At some stage assume that the community ontology 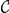 has specified the concept type orderings 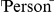 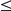 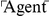, 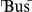 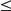 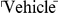 and 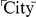 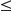 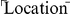 with corresponding injective aspects 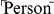 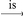 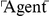, 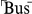 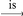 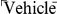 and 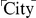 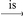 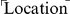. At the next stage it could define a concept type with aspects 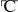 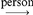 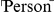, 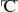 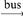 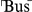 and 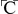 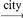 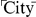, and link it with the reference ontology concept by specifying a connecting aspect 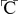 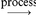 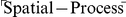 and asserting the facts ‘
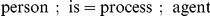
’, ‘
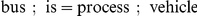
’ and ‘
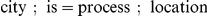
’. In the more expressive ologs with joins (Section 5), the process concept of “going to city by bus” can then be defined as the pullback of the “spatial process” concept: here, the concept type with aspects 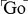 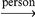 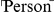, 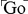 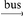 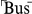 and 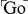 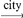 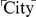 is pulled back along the above injective aspects, resulting in the injective aspect 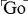 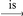 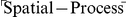 with corresponding concept type ordering 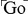 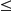 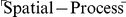. As a result, the concept 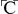 has the new mediating aspect 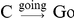, which satisfies the fact ‘
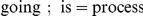
’. In this manner the community ontology 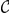 has been enlarged.
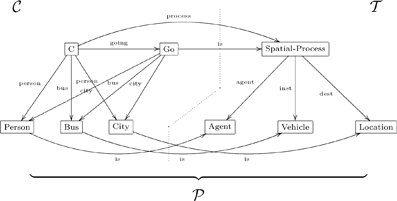



We assume that community ontology 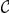 and reference ontology 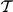 are combined into a portal ontology 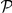 with portal link 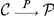 and alignment link 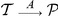. If some other ontology 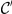 is built up and aligned in the same fashion, then 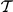 is being used as a common ground, and we have a ‘W’-shaped information system
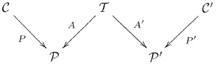
(32)


with portals 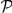 and 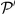 being the final parts. This ‘W’-shaped information system uses the sketch institution Sk for ologs. It can be compared to the ‘W’-shaped information system in [23], which uses the information flow IF institution for (local) logics.

## 5 More expressive ologs I

In this section and the next (5 and 6) we will introduce limits and colimits within the context of ologs. These will allow authors to build ologs that are quite expressive. For example we can declare one type to be the union or intersection of other types. We do not assume mathematical knowledge beyond that of sets and functions, which were loosely defined in Section 2.2. However, the reader may benefit by consulting a reference on category theory, such as [5].

The basic ologs discussed in previous sections are based on the mathematical notion of categories, whereas the olog presentation language we will discuss in this section and the next are based on *general sketches* (see [24]). The difference is in what can be expressed: in basic ologs we can declare types, aspects, and facts, whereas in general ologs we can express ideas like products and sums, as we will see below.

We will begin by discussing layouts, which will be represented categorically by “finite limits”. As usual, the english terminology (layout) is not precise enough to express the notion we mean it to express (limit). Intuitively, a limit can be thought of as a system: it is a collection of units, each of a specific type, such that these units have compatible aspects. These will include types like 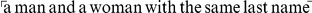. In Section 6 we will discuss groupings, which will be represented by colimits. These will include types like 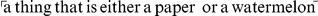.

### 5.1 Layouts

A dictionary might define the word *layout* as something like “a structured arrangement of items within certain limits; a plan for such arrangement.” In other words, we can lay out or specify the need for a set of parts, each of a given type, such that the parts fit together well. This idea roughly corresponds to the notion of limits in category theory, especially limits in the category of sets. Given a diagram of sets and functions, its limit is the set of ways to accordingly choose one element from each. For example, we could have a type 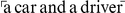, which category-theoretically is a product, but which we are calling a “layout” – a compound type whose parts are “laid out.” Of course, the term layout is insufficient to express the precise meaning of limits, but it will have to do for now. To understand limits, one really only need understand pullbacks and products. These will be the subjects of Sections 5.2 and 5.3, or one can see [5] for more details.

### 5.2 Pullbacks

Given three objects and two arrows arranged as to the left, the pullback is the commutative square to the right
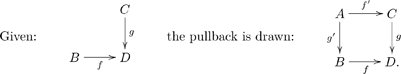
(33)


We write 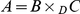 and say “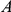 is the pullback of 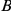 and 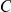 over 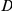.” The question is, what does it signify? We will begin with some examples and then give a precise definition.

#### 5.2.1 Example

We will now give four examples to motivate the definition of pullback. In the first example, (34), both 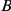 and 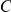 will be subtypes of 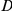, and in such cases the pullback will be their intersection. In the next two examples (35 and 36), only 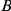 will be a subtype of 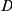, and in such cases the pullback will be the “corresponding subtype of 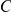
” (as should make sense upon inspection). In the last example (37), neither 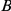 nor 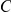 will be a subtype of 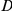. In each line below, the pullback of the diagram to the left is the diagram to the right. The reader should think of the left-hand olog as a kind of problem to which the new box 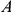 in the right-hand olog is a solution.
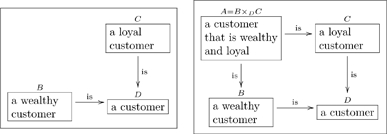
(34)

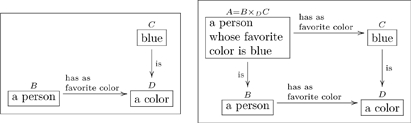
(35)

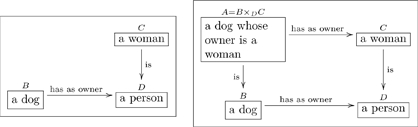
(36)

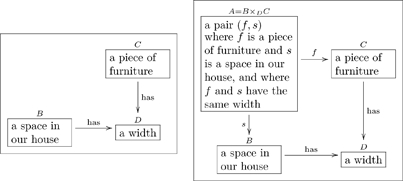
(37)


See Example 5.2.3 for a justification of these, in light of Definition 5.2.2.

The following is the definition of pullbacks in the category of sets. For an olog, the instance data are given by sets (at least in this paper, see Section 3), so this definition suffices for now. See [5] for more details on pullbacks.

#### 5.2.2 Definition

Let 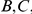 and 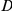 be sets, and let 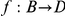 and 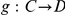 be functions. The *pullback* of 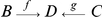, denoted 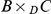, is defined to be the set
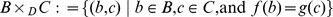



together with the obvious maps 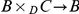 and 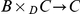, which send an element 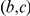 to 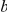 and to 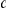, respectively. In other words, the pullback of 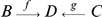 is a commutative square
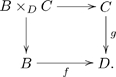
(38)


#### 5.2.3 Example

In Example 5.2.1 we gave four examples of pullbacks. For each, we will consider 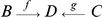 to be sets and functions as in Definition 5.2.2 and explain how the set 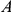 follows that definition, i.e., how its label fits with the set 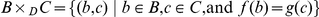.

In the case of (34), the set 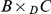 should consist of pairs 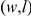 where 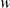 is a wealthy customer, 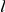 is a loyal customer, and 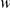 is equal to 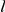 (as customers). But if 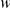 and 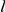 are the same customer then 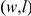 is just a customer that is both wealthy and loyal, not two different customers. In other words, an instance of the pullback is a customer that is both loyal and wealthy, so the label of 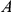 fits.

In the case of (35), the set 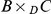 should consist of pairs 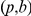 where 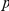 is a person, 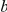 is the color blue, and the favorite color of 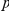 is equal to 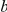 (as colors). In other words, it is a person whose favorite color is blue, so the label of 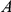 fits. If desired, one could instead label 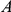 with 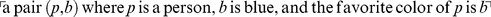.

In the case of (36), the set 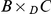 should consist of pairs 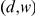 where 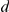 is a dog, 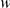 is a woman, and the owner of 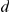 is equal to 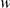 (as people). In other words, it is a dog whose owner is a woman, so the label of 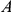 fits. If desired, one could instead label 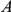 with 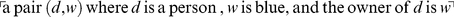.

In the case of (37), the set 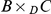 should consist of pairs 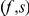 where 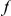 is a piece of furniture, 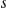 is a space in our house, and the width of 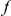 is equal to the width of 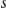. This is fits perfectly with the label of 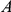.

#### 5.2.4 Using pullbacks to classify

To distinguish between two things, one must find a common aspect of the two things for which they have differing results. For example, a pen is different from a pencil in that they both use some material to write (a common aspect), but the two materials they use are different. Thus the material which a writing implement uses is an aspect of writing implements, and this aspect serves to segregate or classify them. We can think of three such writing-materials: graphite, ink, and pigment-wax. For each, we will make a layout in the olog below:
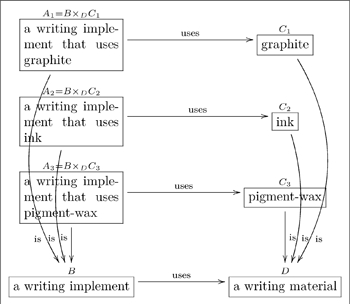
(39)


One could also replace the label of box 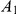 with “a pencil”, the label of box 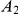 with “a pen”, and the label of box 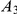 with “a crayon”; in so doing, the layouts at the top would *define* a pencil, a pen, and a crayon to be a writing implement that uses respectively graphite, ink, and pigment-wax.

#### 5.2.5 Building pullbacks on pullbacks

There is a theorem in category theory which states the following. Suppose given two commutative squares
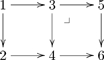
(40)


such that the right-hand square (3,4,5,6) is a pullback. It follows that if the left-hand square (1,2,3,4) is a pullback then so is the big rectangle (1,2,5,6). It also follows that if the big rectangle (1,2,5,6) is a pullback then so is the left-hand square (1,2,3,4). This fact can be useful in authoring ologs.

For example, the type 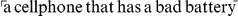 is vague, but we can lay out precisely what it means using pullbacks
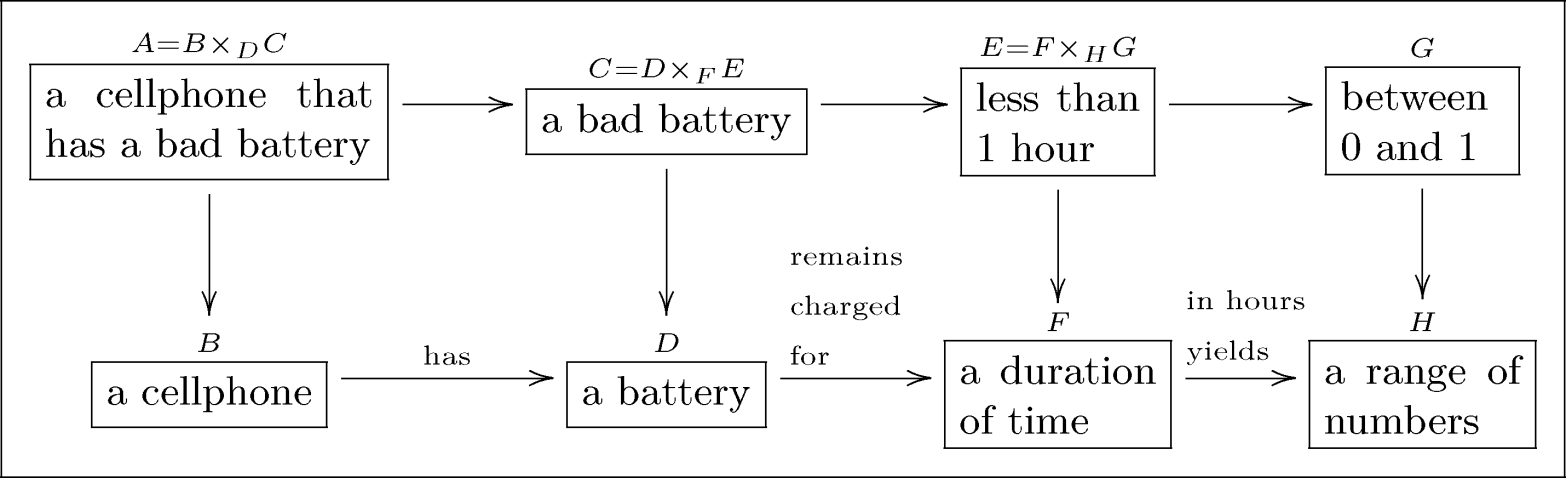
(41)


The category-theoretic fact described above says that since 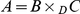 and 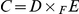, it follows that 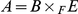. That is, we can deduce the definition “a cellphone that has a bad battery is defined as a cellphone that has a battery which remains charged for less than one hour.” In other words, 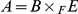.

### 5.3 Products

Given a set of types (boxes) in an olog, one can select one instance from each. All the ways of doing just that comprise what is called the product of these types. For example, if 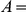 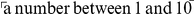  and 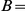 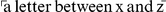 , the product includes a total of 30 elements, including 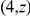. We are ready for the definition.

#### 5.3.1 Definition

Given sets 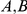, their *product*, denoted 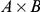, is the set
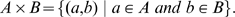
(42)


There are two obvious *projection maps* 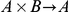 and 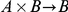, sending the pair 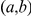 to 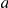 and to 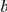 respectively.

#### 5.3.2 Example

In Example 5.2.1, (37) we presented the idea of a piece of furniture that was the same width as a space in the house. What if we say that 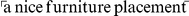 is any space that is between 1 and 8 inches bigger than a piece of furniture? We can use a combination of products and pullbacks to create the appropriate type
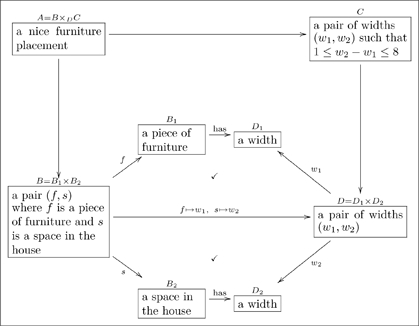
(43)


Here 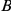 and 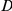 are products and 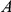 is a pullback. This olog lays out what it means to be “a nice furniture placement” using products. The bottom horizontal aspect 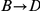 is an example of a map obtained by the “universal property of products”; see Section 5.6.

#### 5.3.3 Products of more (or fewer) types

The product of two sets 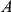 and 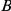 was defined in 5.3.1. One may also take the product of three sets 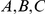 in a similar way, so the elements are triples 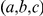 where 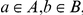 and 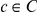. In fact this idea holds for any number of sets. It even makes sense to take the product of one set (just 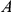) or no sets! The product of one set is itself, and the product of no sets is the singleton set 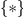. For more on this, see Section 5.5 or [6].

### 5.4 Declaring an injective aspect

A function is called *injective* if different inputs always yield different outputs. For example the function that doubles every integer (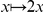) is injective, whereas the function that squares every integer (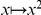) is not because 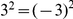. An example of an injective aspect is  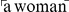 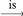 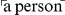  because different women are always different as people. An example of a non-injective aspect is 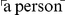 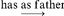 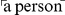  because different people may have the same father.

The easiest way to indicate that an aspect is injective is to use a “hook arrow” as in 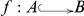, instead of a regular arrow 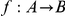, to denote it. For example, the first map is injective (and specified as such with a hook-arrow), but the second is not in the olog:
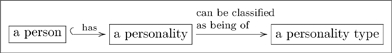
(44)


The author of this olog believes that no two people can have precisely the same personality (though they may have the same personality type).

We include injective aspects in this section because it turns out that injectivity can also be specified by pullbacks. See [25] for details.

### 5.5 Singleton types

A singleton set is a set with one element; it can be considered the “empty product.” In other words if we denote 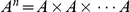 (where 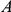 is written 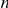 times), then 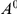 is the empty product and is a singleton set. One can specify that a certain type has only one instance by annotating it with 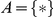 in the olog. For example the olog
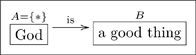
(45)


says that the author considers 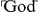 to be single. As a more concrete example, the intersection of 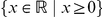 and 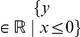 is a singleton set, as expressed in the olog
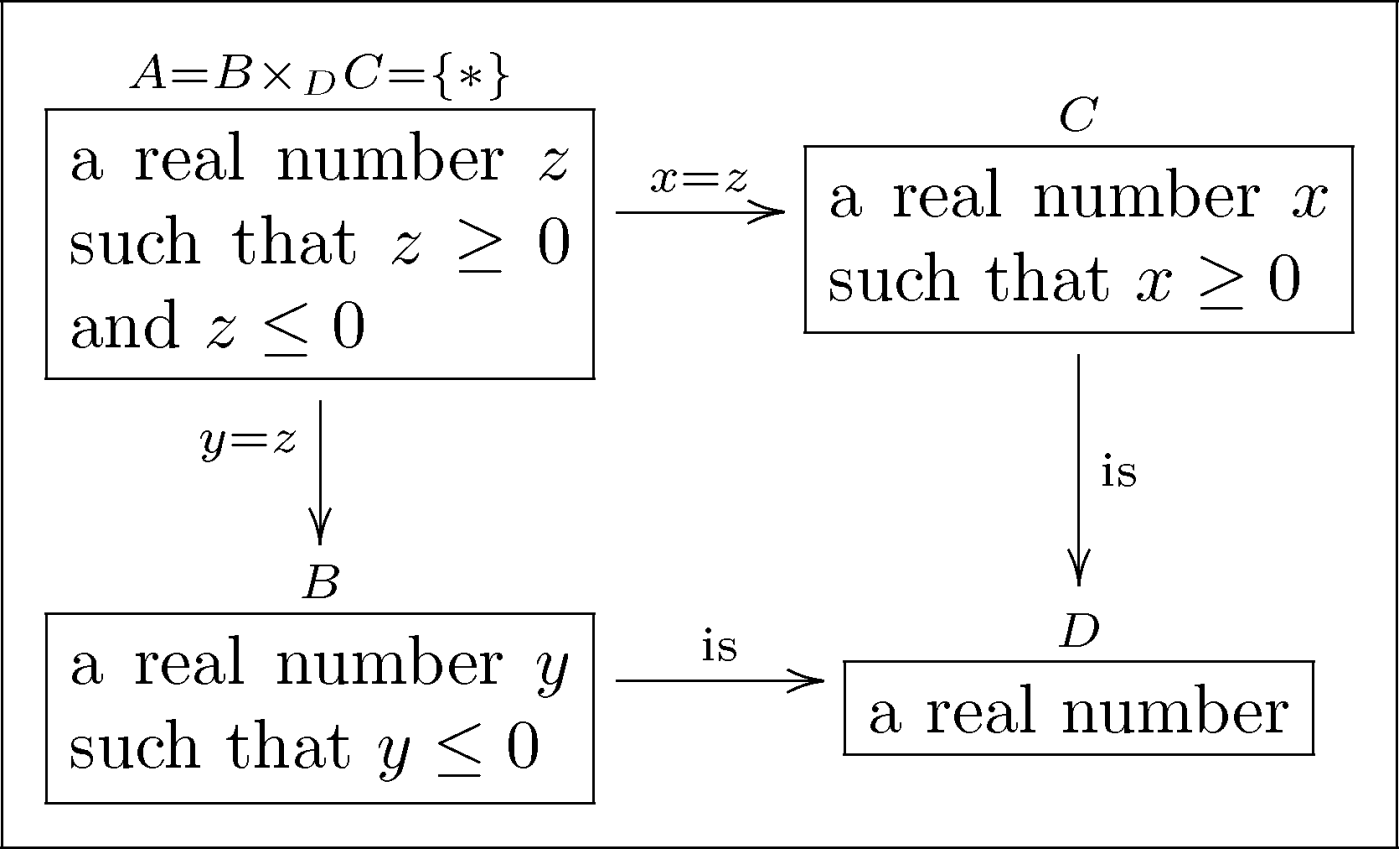
(46)


The fact that 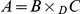 and 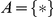 are declared indicates that there is only one possible instance of a real number that is in both 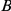 and 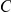.

### 5.6 The universal property of layouts

We cannot do the notion of universal properties justice in this paper, but the basic idea is as follows. Suppose that 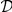 is an olog, that 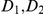 are types in it, and that 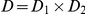 (together with its projection maps 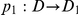 and 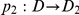) is their product.
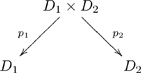
(47)


The so-called universal property of products should be thought of as “an existence and uniqueness” claim in 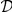. Namely, for any type 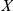 with maps 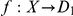 and 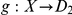, there is exactly one possible map 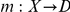 such that the facts 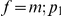 and 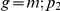 hold.
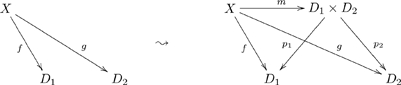
(48)


This may sound esoteric, but consider the following example.

The following olog in similar to the one in Example 5.3.2
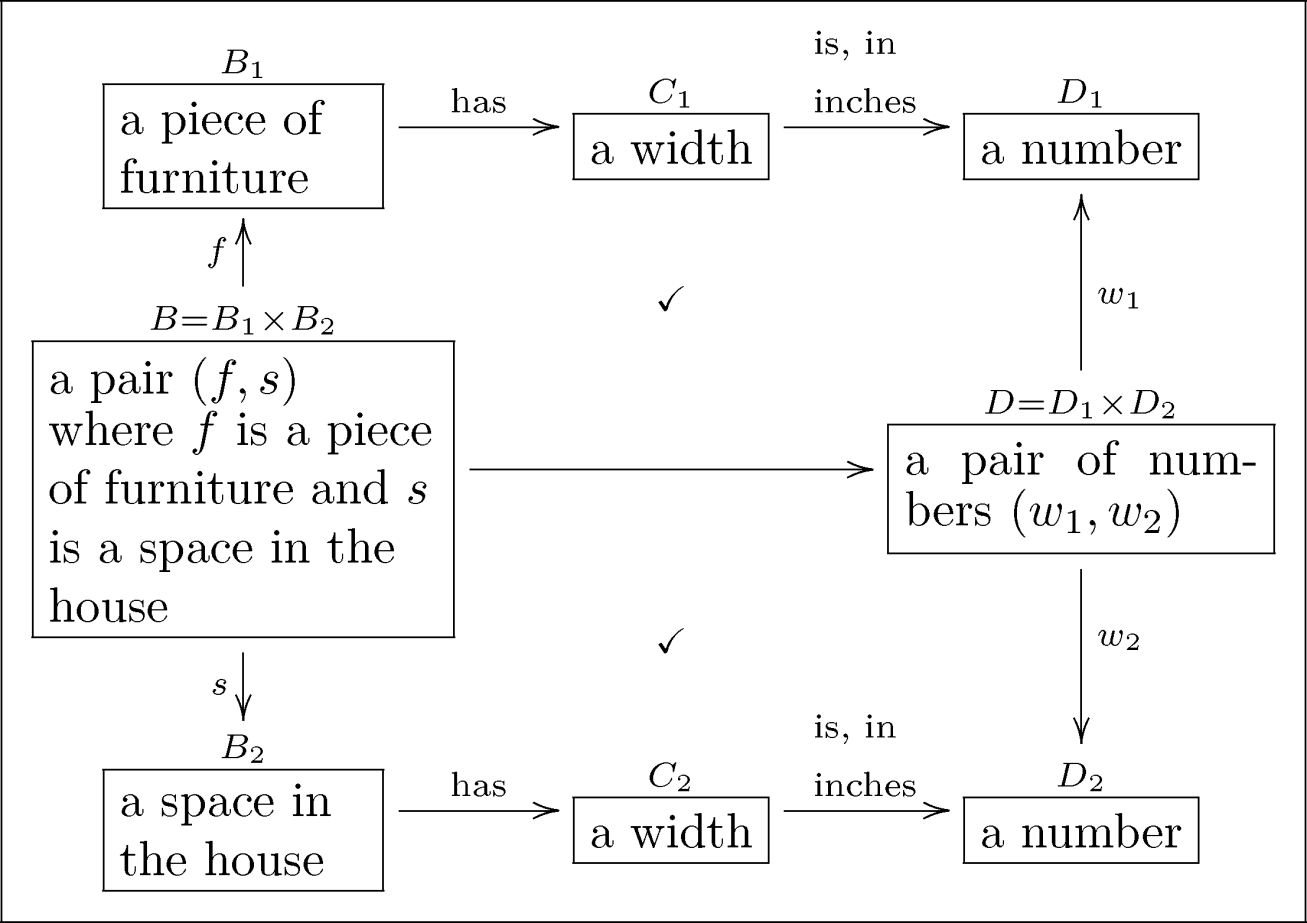
(49)


Here the only unlabeled map is the horizontal one 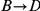; how can we get away with leaving it unlabeled? How does a piece of furniture and a space in the house yield a pair of numbers? The answer is that 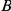 has a map to 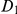 (the path across the top) and a map to 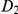 (the path across the bottom), and hence the universal property of products gives a unique arrow 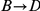 such that the two facts indicated by checkmarks hold. (In terms of (46) and (47) we are using 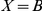.) In other words, there is exactly one way to take a piece of furniture and a space in the house and yield a pair of numbers if we enforce that the first number is the width in inches of the piece of furniture and the second number is the width in inches of the space in the house.

At this point we hope it is clear that the universal property of products is a useful and constructive one. We will not describe the other universal properties (either for pullbacks, singletons, or any colimits); as mentioned above they can be found in [5].

## 6 More expressive ologs II

In this section we will describe various colimits, which are in some sense dual to limits. Whereas limits allow one to “lay out” a team consisting of many different interacting or non-interacting parts, colimits allow one to “group” different types together. For example, whereas the product of 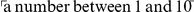 of and 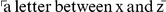 has 30 elements (such as 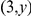), the coproduct of these two types has 13 elements (including 4). Just as “layout” is a too weak a word to capture the essence of limits, “grouping” is too weak a word to capture the essence of colimits, but it will have to do.

We will start by describing coproducts or “disjoint unions” in Section 6.1. Then we will describe pushouts in Section 6.2, wherein one can declare some elements in a union to be equivalent to others. There is a category-theoretic duality between coproducts and products and between pushouts and pullbacks. It extends to a duality between surjections and injections and a duality between empty types and singleton types, the subject of Sections 6.3 and 6.4. The interested reader can see [5] for details.

### 6.1 Coproducts

Coproducts are also called “disjoint unions.” If 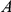 and 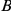 are sets with no members in common, then the coproduct of 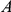 and 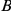 is their union. However, if they have elements in common, one must include both copies in 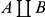 and differentiate between them. Here is a definition.

#### 6.1.1 Definition

Given sets 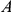 and 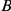, their *coproduct*, denoted 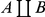, is the set
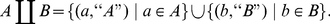
(50)


There are two obvious *inclusion maps* 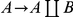 and 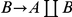, sending 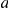 to 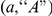 and 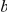 to 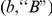, respectively.

If 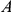 and 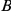 have no elements in common, then the one can drop the “
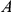
” and “
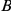
” labels without changing the set 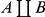 in a substantial way. Here are two examples that should make the coproduct idea clear.

#### 6.1.2 Example

In the following olog the types 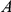 and 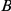 are disjoint, so the coproduct 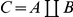 is just the union.
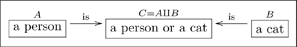
(51)


#### 6.1.3 Example

In the following olog, 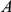 and 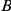 are not disjoint, so care must be taken to differentiate common elements.
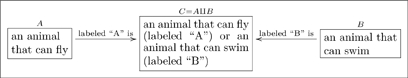
(52)


Since ducks can both swim and fly, each duck is found twice in 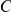, once labeled as a flyer and once labeled as a swimmer. The types 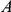 and 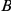 are kept disjoint in 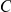, which justifies the name “disjoint union.”

### 6.2 Pushouts

Pushouts can express unions in which an overlap is declared. They can also express “quotients,” where different objects can be declared equivalent. Given three objects and two arrows arranged as to the left, the pushout is drawn as the commutative square to the right
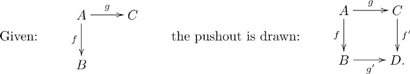
(53)


We write 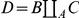 and say “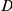 is the pushout of 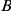 and 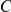 along 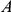.” The question is, what does it signify?

The idea is that an instance of the pushout 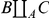 is any instance of 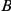 or any instance of 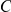, but where some instances are considered equivalent to others. That is, for any instance of 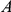, its 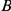-aspect is considered the same as its 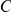-aspect. This is formalized in Definition 6.2.2 after being exemplified in Example 6.2.1.

#### 6.2.1 Example

In each example below, the diagram to the right is the pushout of the diagram to the left. The new object, 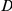, is the union of 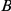 and 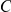, but instances of 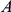 are equated to their 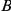 and 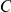 aspects. This will be discussed after the two diagrams.
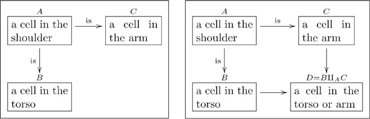
(54)

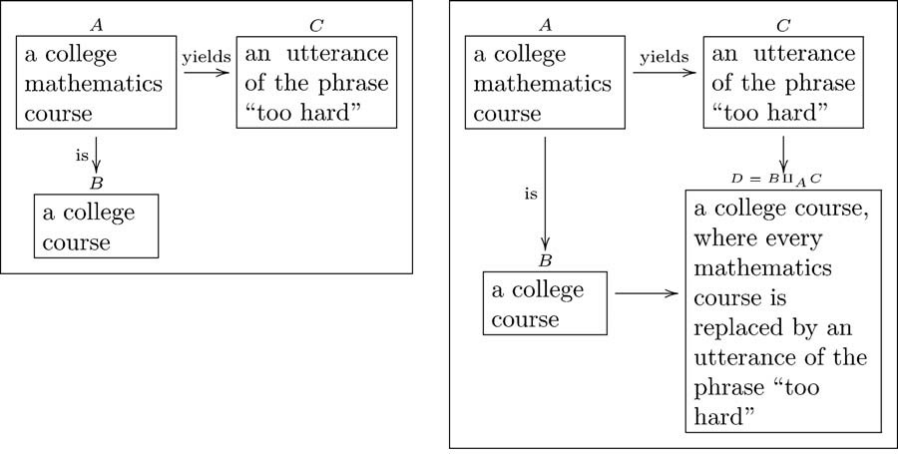
(55)


In the olog (52), the shoulder is seen as part of the arm and part of the torso. When taking the union of these two parts, we do not want to “double-count” the shoulder (as would be done in the coproduct 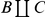, see Example 6.1.3). Thus we create a new type 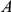 for cells in the shoulder, which are considered the same whether viewed as cells in the arm or cells in the body. In general, if one wishes to take two things and glue them together, the glue serves as 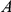 and the two things serve as 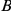 and 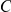, and the union (or grouping) is the pushout 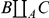.

In the olog (53), if every mathematics course is simply “too hard,” then when reading off a list of courses, each math course will not be read aloud but simply read as “too hard.” To form 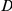 we begin by taking the union of 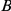 and 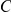, and then we consider everything in 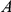 to be the same whether one looks at it as a course or as the phrase “too hard.” The math courses are all blurred together as one thing. Thus we see that the power to equate different things can be exercised with pushouts.

#### 6.2.2 Definition

Let 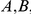 and 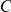 be sets and let 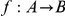 and 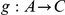 be functions. The *pushout* of 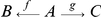, denoted 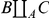, is the quotient of 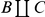 (see Definition 6.1.1) by the equivalence relation generated by declaring 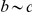 (i.e., 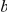 is equivalent to 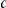) if: 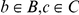, and there exists 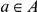 with 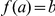 and 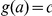.

### 6.3 Declaring a surjective aspect

A function 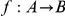 is called *surjective* if every value in 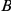 is the image of something in the domain 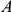. For example, the function which subtracts 1 from every integer (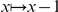) is surjective, because every integer has a successor; whereas the function that doubles every integer (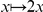) is not surjective because odd numbers are not mapped to. The aspect is 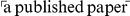 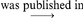 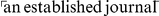 is surjective because every established journal has had at least one paper published in it. The aspect is 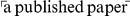 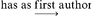 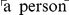 is not surjective because not every person is the first author of a published paper.

The easiest way to indicate that an aspect is surjective is to denote it with a “two-headed arrow” as in 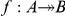. For example, the second map is surjective (and indicated with a two-headed arrow) in the olog
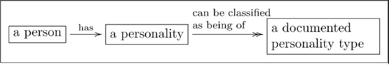
(56)


Here the first aspect is not considered surjective, presumably because the author imagines personalities had by no person.

We include surjective aspects in this section because it turns out that surjectivity can also be specified by pushouts. See [26] for details.

### 6.4 Empty types

The empty set is a set with no elements; it can be considered the “empty coproduct.” In other words if we denote 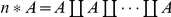 (where 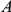 is written 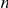 times), then 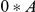 is the empty coproduct and is the empty set. One can declare a type to be empty by annotating it with 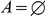 in the olog.
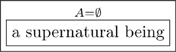
(57)


says that the set of supernatural beings is empty. As a more concrete example, the intersection of positive numbers and negative numbers is empty, as expressed in the olog
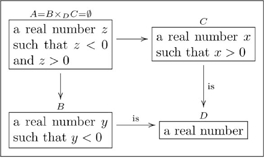
(58)


### 6.5 Images

In what remains of Section 6, we will discuss how the ideas of this section and the previous (Section 5) can be used together to create quite expressive ologs. First we will discuss how each aspect 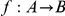 has an “image,” the subset of 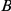 that are “hit” by 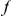. Then, in Sections 6.6 and 6.7, we will discuss how ologs can express all primitive recursive functions and many other mathematical concepts. Consider the olog 
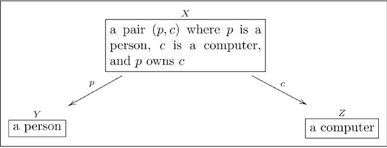
(59)


Some people own more than one computer, and some computers are owned by more than one person. Some computers are not owned by a person, and some people do not own a computer. The purpose of this section is to show how to use ologs to capture ideas such as “a person who owns a computer” and “a computer that is owned by a person”. These are called the images of 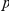 and 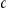 respectively.

Every aspect has an image, and these are quite important for human understanding. For example the image of the map  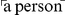 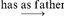 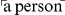  is the type 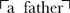. In other words, a father is defined to be a person 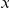 for which there is some other person 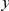 such that 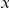 is the father of 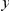.

The image of a function 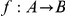 is a commutative diagram (fact)
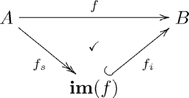
(60)


where 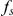 is surjective and 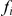 is injective (see Sections 6.3 and 5.4). We indicate that a type is the image of a map 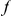 by annotating it with **Im** 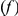, as in follow olog:
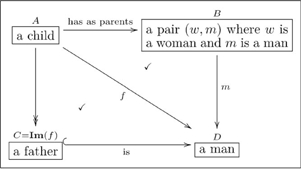
(61)


Hopefully it is also clear that 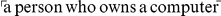 and 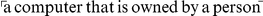 are the images of 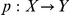 and 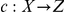 (respectively) in Olog (57).

Using the label **Im** 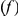 is the easiest way to indicate an image, although one can also do so categorically using limits and colimits. See [6, Chapter VIII] for details.

### 6.6 Application: Primitive recursion

We have already seen how ologs can be used to express a conceptual understanding of a situation (all the ologs thus far exemplify this idea). In this section we hope to convince the reader that ologs are also able to express certain computations. In particular we will show by example that primitive recursive functions (like factorial or fibonacci) can be expressed by ologs. In this way, we can to put computation and knowledge representation together into the same framework. It would be quite valuable to strengthen this connection by showing that Ologs (or an extension thereof) can express any recursive function (i.e., simulate Turing machines). This is an open research possibility.

*Example* 6.6.1. In this example we will present an olog that can represent the “Factorial function,” often denoted 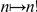, where for example the factorial of 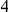 is 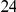. Recall that a *natural number* is any nonnegative whole number: 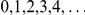.
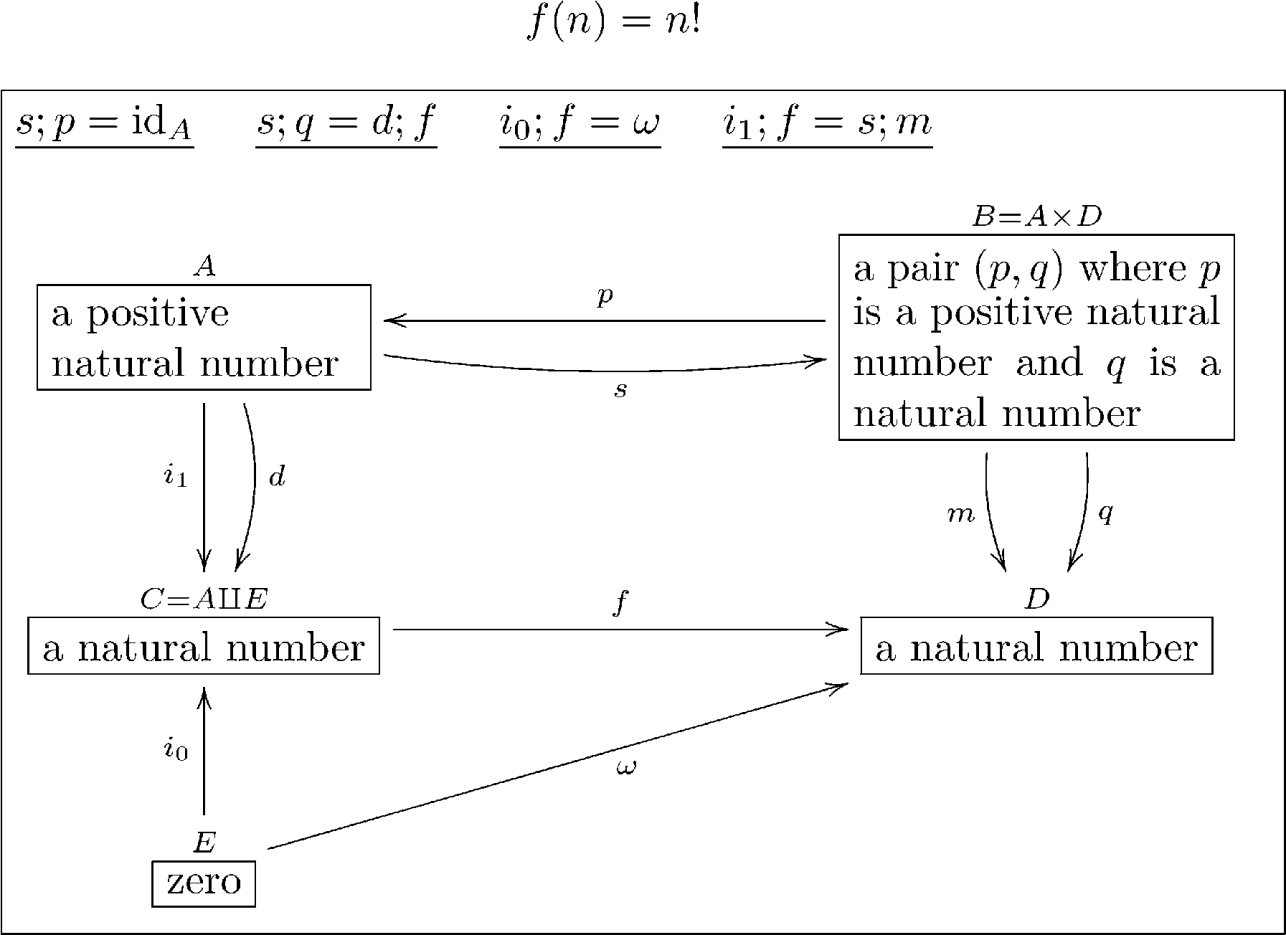
(62)


The idea of this olog is to convey the factorial function as follows. A natural number is either zero or positive. Every positive natural number 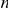 has a decrement, 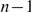. The factorial of zero is 1. The factorial of a positive number 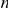 is obtained by multiplying 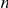 by the factorial of 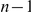.

To more explicitly describe the above olog, we must describe its intended instances. Hopefully the instances of each type (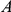 through 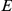) are self-explanatory, so we will describe the grouping, the layout, the aspects, and the facts. The set of natural numbers is the disjoint union of zero and the set of positive natural numbers and the maps 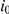 and 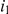 are the inclusions into the coproduct, which explains the grouping 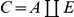. The layout 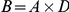 is self-explanatory, and the maps 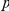 and 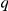 are the projections from the product. The map 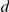 is the decrement map 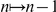, the map 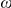 sends 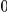 to 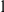, the map 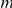 is multiplication 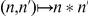. Once 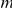, 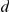, and 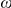 are so-defined, the first two facts (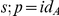 and 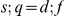) specify that 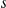 sends 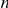 to the pair 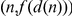, and the second two facts specify that 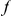 sends 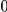 to 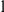 and sends a positive number 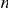 to 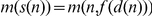, i.e., 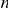 goes to the product 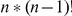.

The above olog defines the factorial function (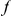) in terms of itself, which is the hallmark of primitive recursion. Note, however, that this same olog can compute many things besides the factorial function. That is, nothing about the olog says that the instances of 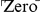 is the set 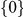, that 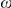 sends 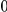 to 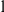, that 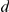 is the decrement function, or that 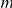 is multiplication – changing any of these will change 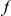 as a function. For example, the same olog can be used to compute “triangle numbers” (e.g. f(4) = 1+2+3+4 = 10) by simply changing the instances of 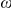 and 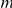 in the obvious ways (use 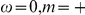 rather than 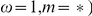). For a radical departure, fix any forest (set of graphical trees) 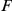, let 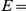 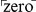  represent its set of roots, 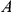 the other nodes, 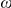 the constant 0 function, 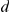 the parent function, and 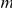 sending 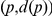 to 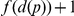. Then for each tree in 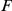 and each node 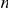 in that tree, the function 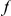 will send 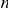 to its height on the tree.

Primitive recursion is a powerful technique for deriving new functions from the repetition of others using a kind of “while loop.” The general form of primitive recursive functions can be found in [27], and it is not hard to imitate Example 6.6.1 for the general case.

### 6.7 Application: Defining mathematical concepts

In this subsection we hope to convince the reader that many mathematical concepts can be defined by ologs. This should not seem like much of a stretch: ologs describe relationships between sets, so we rely on the maxim that all of mathematics can be formulated within set theory. To make the idea explicit, however, we will recall the definition of pseudo-metric space (in 6.7.1) and then provide an olog with the same content (in 61).

#### 6.7.1 Definition

Let 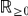 denote the set of non-negative real numbers. A *pseudo-metric space* is a pair 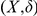 where 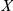 is a set and 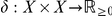 is a function with the following properties for all elements 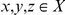:

1. 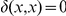;

2. 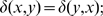 and

3. 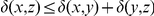.
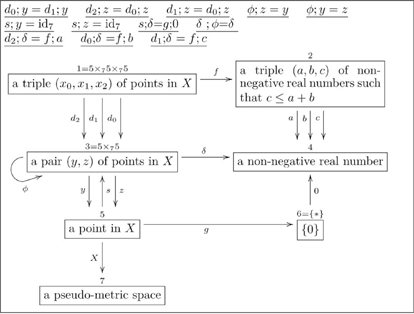
(63)


As long as the instances for the right-hand side of the olog are mathematically correct (i.e., we assign 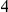 the set of non-negative real numbers), this olog has the same content as Definition 6.7.1. One can use ologs to define usual metric spaces (in which Property (1) in Definition 6.7.1 is strengthened), but it would have taken too much space here.

It should be clear that ologs provide a more precise and explicit description of any concept, relying less on the grammar of English and more on the mathematical “grammar” of sets and functions. Assumptions are exposed as all the working parts of an object need to be explicitly documented. Thus an olog is likely to be instantly readable by a theorem prover such as Coq ([28]), at least if one creates the olog within an appropriate Olog-Coq interface API. Moreover, various parts of this olog may be reusable in other contexts, and hence connect pseudo-metric spaces into a web of neighboring definitions and theorems.

In fact, once a corpus of mathematics has been written in olog form, evidence of conjectures not yet proven could be written down as instance data. For example, one could record every known prime as instances of a type and a machine could automatically check that Goldbach’s conjecture (written as an olog containing as a type) holds for all example “so far.” With definitions, theorems, and examples all written in the same computer-readable language of ologs, one may hope for much more advanced searching and knowledge retrieval by humans. For example, one could formulate very precise questions as database queries and use SQL on the database corresponding to a given olog (see Section 3.2).

## 7 Further directions

Ologs are basically categories which have text labels to explain their intended semantic. As such there are many directions to explore ranging from quite theoretical to quite practical. Here we consider three main classes: extending the theory of ologs, studying communication with ologs, and implementing ologs in the real world.

### 7.1 Extending the theory of ologs

In this paper we began by discussing basic ologs, which are rich enough to capture the semantic of many situations. In Sections 5 and 6 we added more expressivity to ologs to allow one to encode ideas such as intersections, unions, and images. However, ologs could be even more expressive. One could add “function types” (also known as exponentials); add a “subobject classifier type,” which could allow for negation and complements as well as power-sets; or even add fixed sets (like the set of Strings) to the language of ologs. This is not too hard (using sketches, see [24]); the reason we did not include them in this paper was more because of space than any other reason.

Another generalization would be to allow the instances of an olog to take values in a category other than 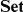. For example, one could have an instance-space rather than an instance-set, e.g. it is clear that the instances of the type ⌜a point on the unit circle⌝ constitute a topological space. One could similarly argue that the instances of the type 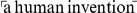 have a topology or metric as well (e.g. as an invention, the cellphone is closer to the telephone than it is to artificial flavoring). Instance data on an olog 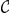 corresponds to a functor 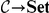 in this paper, but it is quite easy to replace 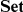 with a different category such as 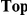 (the category of topological spaces), and this may have interesting uses in data modeling.

In Section 6.7, we explicitly showed that pseudo-metric spaces (and we stated further that metric spaces) can be presented by ologs. It would be interesting to see if theorems could also be proven entirely within the context of ologs. If so, a teacher could first sketch a mathematical proof as a small or sparse olog 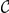, and then use a functor 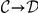 to rigorously “zoom in” on that proof so that the sketch becomes a full-fledged proof (as the maps in 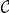 are factored into understandable units in 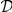).

If ologs are to be viable venues in which to discuss results in mathematics, then they should be capable of describing all recursion, not just primitive recursion (as in Section 6.6). We do not yet have an understanding for how this can be done. If recursion can be fully defined with the ologs described above, it would be interesting to see it written out; if not, it would be interesting to understand what basic idea could be gracefully added to ologs so that recursion becomes expressible.

In a different direction, one could test the expressive power of ologs by defining simple games, like Tic Tac Toe or Chess, using ologs. It would be impressive to define a vocabulary for writing games and a program which could automatically convert an olog-defined game into a playable computer game. This would show that the same theory that we have seen express ideas about fatherhood and factorials can also be used to invent games and program computers.

### 7.2 Studying communication with ologs

As discussed in Section 4, ologs can be connected by functors into networks that are not just 2-way, but 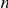-way. These communication networks should be studied: what kinds of information can pass, how reliable is it, how quickly can it spread, etc. This may be applicable in fields from economics to psychology to sociology. Such research may use results from established mathematics such as Network Coding Theory (see [29]).

Spivak and coauthor Mathieu Anel are preparing for publication the results of their mathematical description of how two or more entities (described as ologs) can communicate new ideas (not just new instance data) to each other. It would be interesting to see how well this “communication protocol” works in practice, and whether it can be theoretically automated. Furthermore, this communication protocol and any theoretical automation of it should be implemented on a computer to see if different database schemas can be meaningfully integrated with minimal human assistance.

It may be possible to train children to create ologs about their interests or about a given lesson. These ologs would show how the child actually perceives something, which would probably be fascinating. By our experience and that of people we have taught, the process of building an olog usually leads to a clarification of the concepts involved. Moreover, a class project to connect the ologs of different students and between the students and the teacher, may have excellent pedagogical benefits.

Finally, it may be interesting to study “local truth” vs. “global truth” in a network of ologs. Functorial connections between ologs can allow for translation of ideas between members of a group, but there may be ideas which do not extend globally, just as a Möbius band does not admit a global orientation. That is, given three parties on the Möbius band, any pair can agree on a compass orientation, but there is no choice that the three can simultaneously agree on. Similarly, whether or not it is possible to construct a global language which extends all the existing local ones could be determined if these local languages and their connections were entered into a computer olog system.

### 7.3 Implementing ologs in the real world

Once ologs are implemented on computers, and once people learn how to author good ologs, much is possible. One advantage comes in searching the information space. Currently when we search for a concept (say in Google or on our hard drive), we can only describe the concept in words and hope that those words are found in a document describing the concept. That is, search is always text-based. Better would be if the concept is meaningfully interconnected in a web of concepts (an olog) that could be navigated in a meaningful (as opposed to text-based) way.

Indeed, this is the semantic web vision: When internet data is machine-readable, search becomes much more powerful. Currently, we rely on RDF scrapers that scour web pages for 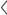
subject, predicate, object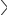 sentences and store them in RDF format, as though each such sentence is a fact. Since people are inputting their data as prose text, this may be the best available method for now; however, it is quite inaccurate (e.g. often 15% of the facts are wrong, a number which can lead to degeneration of deductive reasoning – see [30]). If ideas could be put on the internet such that they compatibly made sense to both human and computer, it would give a huge boost to the semantic web. We believe that ologs can serve as such a human-computer interface.

While it is often assumed that because we all speak the same language we all must mean the same things by it, this is simply not true. The age-old question about whether “blue for me” is the same as “blue for you” is applicable to every single word and idiom in our language. There is no easy way to sync up different people’s perceptions. If communication is to be efficient, agreements must be fairly explicit and precise, and this precision demands a rigor that is simply unavailable in English prose. It is available in a network of ologs (as described in Section 4).

For example, the laws of the United States are hopelessly complex. Residents of the US are required to obey the laws. However, unlike the rules of the Scholastic Aptitude Test (SAT), which take 10 minutes for the proctor to read aloud, the laws of the US are never really expressed – the most important among them are hopefully picked up by cultural osmosis. If an olog was created which had enough detail that laws could be written in that format, then a woman could research for herself whether her landlord was required to fix her refrigerator or whether this was her responsibility. It may prove that the olog of laws is internally inconsistent, i.e., that it is impossible for a person to satisfy all the laws – such an analysis, if performed, could fundamentally change our outlook on the legal system.

The same goes for science; information written up in articles is much less accessible than information that is entered into an ontology. However, the dream of a single universal ontology is untenable ([31]). Instead we must allow each lab or institute to create its own ontology, and then require citations to be functorial olog connections, rather than mere silo-to-silo pointers. Thus, a network of ologs should be created to represent the understanding of the modern scientific community as a multi-faceted whole.

Another impetus for a scientist to write an olog about the study at hand is that, once an olog is made, it can be instantly converted to a database schema which the scientist can use to input all the data pertaining to this study. Indeed, if some data did not fit within this schema, then the olog must have been insufficient to begin with and should be modified to fully describe the experiment. If scientists work this way, then the separation between them and database modelers can be reduced or eliminated (the scientist assumes the database modeling role with little additional burden). Moreover, if functorial connections are established between the ologs of different labs, then data can be meaningfully shared along those connections, and ideas written in the language of one lab’s olog can be translated automatically into the language of the other’s. The speed and accuracy of scientific research should improve.
